# Allogeneic “Zombie Cell” as Off‐The‐Shelf Vaccine for Postsurgical Cancer Immunotherapy

**DOI:** 10.1002/advs.202307030

**Published:** 2024-01-26

**Authors:** Bo Li, Ping Zhang, Junlin Li, Rui Zhou, Minglu Zhou, Chendong Liu, Xi Liu, Liqiang Chen, Lian Li

**Affiliations:** ^1^ Key Laboratory of Drug‐Targeting and Drug Delivery System of the Education Ministry and Sichuan Province Sichuan Engineering Laboratory for Plant‐Sourced Drug and Sichuan Research Center for Drug Precision Industrial Technology West China School of Pharmacy Sichuan University Chengdu 610041 China; ^2^ NMPA Key Laboratory for Technical Research on Drug Products In Vitro and In Vivo Correlation Sichuan Institute for Drug Control Chengdu 611730 China

**Keywords:** biomedical engineering, cell delivery, localized therapy, postsurgical immunotherapy, whole tumor cell vaccine

## Abstract

Allogeneic tumor cell vaccines provide off‐the‐shelf convenience but lack patient specificity due to heterogeneity in tumor antigens. Here, allogeneic tumor cell corpses are converted into “zombie cells” capable of assimilating heterogeneous tumor by seizing cancer cells and spreading adjuvant infection. This causes pseudo‐oncolysis of tumors, transforming them into immunogenic targets for enhanced phagocytosis. It is shown that in postoperative tumor models, localized delivery of premade “zombie cells” through stepwise gelation in resection cavity consolidates tumor surgery. Compared to analogous vaccines lacking “seizing” or “assimilating” capability, “zombie cell” platform effectively mobilizes T cell response against residual tumors, and establishes immunological memory against tumor re‐challenge, showing less susceptibility to immune evasion. Despite using allogeneic sources, “zombie cell” platform functions as generalizable framework to produce long‐term antitumor immunity in different tumor models, showing comparable effect to autologous vaccine. Together, with the potential of off‐the‐shelf availability and personalized relevance to heterogenous tumor antigens, this study suggests an alternative strategy for timely therapy after tumor surgery.

## Graphical abstract

A stepwise gelation platform is designed to deliver “zombie cells” to tumor resection cavity. These “zombie cells”, capable of assimilating heterogeneous tumor by seizing cancer cells and triggering pseudo‐oncolysis effect by spreading adjuvant infection, can successfully mobilize specific T cell response, thus combining the benefits of off‐the‐shelf availability and personalized relevance to patients’ heterogenous antigens.

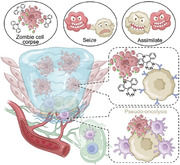

## 1 Introduction

Tumor vaccines using defined antigens have long been employed to treat cancers by mobilizing host immunity.^[^ ^1^ ^]^ However, the heterogeneity and complexity of antigens across patients as well as the high expense of identifying patient‐specific antigen mutations impede their widespread clinical use.^[^ ^2^ ^]^ To overcome these limitations, autologous tumors have been used to produce whole tumor cell vaccines (namely personalized cancer vaccines).^[^ ^3^ ^]^ These encompass the full repertoire of potential antigens and can elicit immune responses against the unique antigen profile of an individual's cancer, conferring much less susceptibility to tumor immune evasion.^[^ ^4^ ^]^

Due to low immunogenicity of inactivated tumor cells alone, adjuvant approaches have been explored for autologous cancer vaccines, including co‐delivering whole tumor cells or lysates with immunostimulatory molecules like granulocyte macrophage colony stimulating factor (GM‐CSF), cytosine‐phosphodiester‐guanine (CpG) oligodeoxynucleotide, and interleukin (IL‐2).^[^ ^5^ ^]^ Additionally, gene transfection to directly modify tumor cells have been studied, such as integrating retroviruses to engineer the cells to self‐produce GM‐CSF.^[^ ^6^ ^]^ Previously, we generated a self‐adjuvanting vaccine by inducing programmed oncolysis of surgically resected tumor‐derived cells, enabling continuous emission of endogenous “eat me” and “danger” signals and durable, antigen‐specific immunity against postoperative tumor residuals.^[^ ^7^ ^]^ However, we also found that in practice, the time‐consuming procedures between tumor acquisition and vaccination contradict the narrow time window for postsurgical tumor intervention.^[^ ^8^ ^]^ Moreover, bioengineering of precious autologous tumor cells requires highly trained personnel, which increases costs, delays treatment, and causes unpredictable effectiveness.^[^ ^9^ ^]^ Therefore, an ideal tumor cell vaccine should be an “off‐the‐shelf” product conductive to timely treatment of residual tumors post‐surgery, while yet being a “personalized” product overcoming the low antigenic relevance of allogeneic vaccines to a patient's heterogenous tumor.^[^ ^10^ ^]^

**Figure 1:**
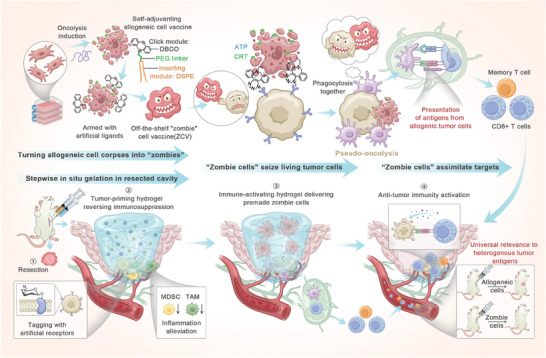
Schematic illustration of an off‐the‐shelf allogeneic “zombie cell” vaccine to consolidate tumor surgery. “Zombie cell” vaccine uses premade allogeneic cancer cell corpses fabricated ex vivo. The cells are treated with clinical‐grade oncolytic peptide of LTX‐315, and inserted with surface‐anchor of DSPE‐PEG_2k_‐DBCO, resulting in cell corpses emitting oncolysis‐induced immunoadjuvant and empowered with artificially ligands. The delivery strategy consists of stepwise in situ gelation in tumor resection cavity. A tumor‐priming hydrogel is injected first to alleviate inflammation for local immunosuppression reversal and glycoengineer residual tumor cells with artificial azido receptors. In sequence, an immunoactivatory hydrogel delivering “zombie cells” is then injected, allowing bioorthogonal conjugation to residual tumor cell surfaces. Analogous to zombie behavior of the walking dead preying on the living and assimilating the heterogeneous, “zombie cell” vaccine leverages the cell corpses to seize residual tumor cells and spread adjuvant properties. This induces pseudo‐oncolysis of the allogeneic tumor cell targets, converting them into immunogenic stimuli for activating specific anti‐tumor immunity.

Here, we have designed an off‐the‐shelf vaccine utilizing “zombie cells” to consolidate tumor surgery. In this design, “zombie cell” corpses succumbing to oncolysis, emitting adjuvanting signals, and artificially armed with chemical ligands can be manufactured in batches ahead of time from readily available sources of allogeneic cancer cells that have been adapted for growth and engineering in culture. The delivery strategy consists of stepwise in situ gelation in tumor resection cavity. Immediately after surgery, the first hydrogel is injected locally to reverse the immunosuppression by alleviating the surgical stress‐induced inflammation in residual tumors, and simultaneously enable metabolic glycoengineering to chemically tag living tumor cells with artificial receptors, together priming the tumor residuals to prepare for “zombie cells”. Sequentially after the first hydrogel degrades, the second hydrogel is injected into the resection cavity, where it immediately forms a scaffold to gradually release the “zombie cells” that eventually anchor onto the surface of residual tumor cells through bioorthogonal conjugation. Upon cell‐cell contact, “zombie cells”, acting like cell‐surface factories that massively produce damage‐associated molecular patterns (DAMPs), camouflage the tumor cells in a pseudo‐oncolysis state. This leads to the recruitment of antigen‐presenting cells and facilitates the indiscriminate phagocytosis of both “zombie cells” and targeted tumor cells, subsequently activating immune response against tumor residuals. Akin to a zombie flick where the walking dead prey on humans and spread their conditions through contacts, we term this strategy “zombie cell” vaccine (ZCV) because it closely resembles three features of a zombie: 1) ZCV is essentially cell corpses succumbing to oncolysis; 2) ZCV has the capability to “seize” living tumor cells; and 3) ZCV assimilates the heterogeneous targets through spreading adjuvant infections and causing pseudo‐oncolysis (**Figure** **1**).

## 2 Results

### 2.1 Zombie Cells Seize Tumor Cells

**Figure 2:**
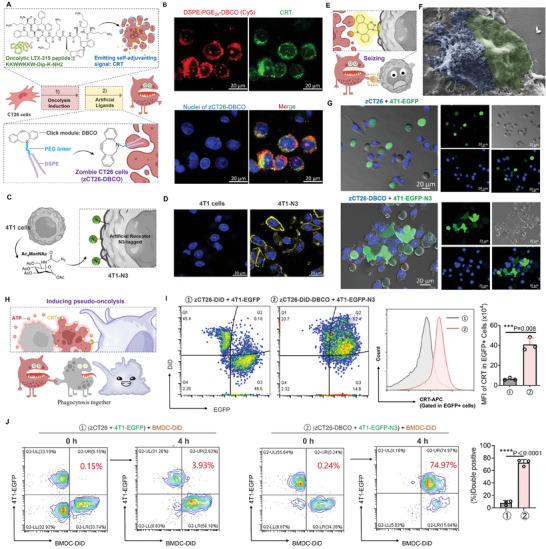
Zombie cells seize tumor cells and induce pseudo‐oncolysis. A) Illustration of two‐step preparation of zCT26‐DBCO via oncolysis induction and artificial ligand insertion. B) Fluorescence image of zCT26‐DBCO. Red, DSPE‐PEG_2k_‐Cy5; Green, CRT; Blue, nuclei of cell corpses; Scale bar, 20 µm. C) Illustration of azido tagging of 4T1 cells by glucometabolic engineering. D) Fluorescence image of unmodified 4T1 cells or 4T1‐N_3_ cells stained with DBCO‐Cy5. Yellow, DBCO; Blue, cell nuclei; Scale bar, 20 µm. E) Schematic diagram of zCT26‐DBCO seizing 4T1‐N_3_ via bioorthogonal reaction. F) Scanning electron microscopy images showing the anchoring of zCT26‐DBCO (blue) onto 4T1‐N_3_ (green) after 1 h mixture. G) Confocal microscopy images of zCT26‐DBCO binding to 4T1‐EGFP‐N_3_ after 1 h mixture. Blue, nuclei of zCT26‐DBCO; Green, EGFP expressed by 4T1 cells; Grey, bright field; Scale bar, 20 µm. H) Illustration of “zombie cells” infecting tumor cells with adjuvanting properties. I) Flow cytometry scatter plot representation of zCT26‐DiD‐DBCO and 4T1‐EGFP‐N_3_ after 1 h mixture (left panel), and quantification of CRT fluorescence intensity gated within EGFP^+^ cell clusters (right panel). J) Flow cytometry analysis of phagocytosis of EGFP‐transfected 4T1 cells by DiD‐labeled BMDCs (BMDC‐DID). Following 1 h premixture of zCT26+4T1‐EGFP, or zCT26‐DBCO+4T1‐EGFP‐N_3_, stimulated BMDC‐DiD were added to allow for 4 h phagocytosis. Data are shown as mean ± SD. ^***^ *P* < 0.001, ^****^ *P* < 0.0001.

To demonstrate the proof‐of‐concept, postoperative model of immunocompetent mouse bearing an orthotopic 4T1 murine breast tumor was selected.^[^ ^11^ ^]^ Meanwhile, an allogeneic cell line, CT26, derived from murine colon carcinoma, was utilized to fabricate “zombie cells” designated as zCT26‐DBCO. Generation of zCT26‐DBCO involved two steps: inducing oncolysis and inserting artificial ligands. As illustrated in **Figure** **2**A, CT26 cells were first treated with a clinical‐grade oncolytic peptide of LTX‐315^[^ ^12^ ^]^ that closely recapitulates the cationic and amphipathic nature of oncolytic virus. Then, using a similar cell‐surface decoration method as previously reported,^[^ ^13^ ^]^ the anchor of dibenzocyclooctylenyl (DBCO)‐bearing, two‐tailed lipids [1,2‐distearoyl‐*sn*‐glycero‐3‐phosphoethanolamine‐*N*‐amino(polyethylene glycol)_2000_‐DBCO (DSPE‐PEG_2k_‐DBCO)] were inserted into the lipid bilayer of the membrane of CT26 cell corpses succumbing to oncolysis. As a result of oncolysis, the cell corpses exhibit typical features of immunogenic cell death that implicated continuous surface exposure of calreticulin (CRT) and extracellular release of adenosine triphosphate (ATP) over time (Figure S1, Supporting Information). These DAMPs can operate on a series of receptors expressed by dendritic cells (DCs) to stimulate the antigen presentation to T cells.^[^ ^14^ ^]^ To characterize the resulting zCT26‐DBCO, DSPE‐PEG_2k_‐DBCO was substituted by fluorescent DSPE‐PEG_2k_‐Cy5, and immunoactivatory CRT was fluorescently labeled. As evidenced by confocal microscopy imaging (Figure 2B), the strong fluorescence of both Cy5 and CRT marker indicated that zCT26‐DBCO had effective surface anchoring of click module (DBCO) via the two‐tailed lipids (DSPE‐PEG_2k_ derivative), while substantially emitting adjuvanting DAMPs.

Having armed “zombie cells” with artificial ligands (DBCO), we next tagged 4T1 tumor cells with complementary receptors (azido) for bioorthogonal click reaction (Figure 2C). Adding unnatural sugar 1,3,4‐*O*‐acetyl‐*N*‐azidoacetylmannosamine (Ac_4_ManNAz) to cells is a well‐established approach to enable metabolic glycoengineering to install azido‐sialic acids on their surfaces via the sialic acid biosynthesis pathway.^[^ ^15^ ^]^ Following the treatment of 4T1 cells with Ac_4_ManNAz, cell‐surface azido was detected by DBCO‐terminated fluorophore (DBCO‐Cy5). Compared to bare binding to unmodified 4T1 cells, DBCO‐Cy5 showed a surface‐bound ring pattern surrounding Ac_4_ManNAz‐treated cells (Figure 2D), indicating the successful expression of azido groups on engineered 4T1 cells (herein denoted as 4T1‐N_3_).

We next investigated whether the artificial bioorthogonal engineering,^[^ ^16^ ^]^ enhanced the interaction of CT26 “zombie cells” for 4T1 tumor cells (Figure 2E). As evidenced by scanning electron microscopy, zCT26‐DBCO with severe membrane lysis and surface perforations could adhere onto the 4T1‐N_3_ with intact membranes (Figure 2F). To further confirm the cell‐cell contacts by confocal microscopy imaging, the nucleus of zCT26‐DBCO was stained blue, and azido‐tagging 4T1 cells were transfected with enhanced green fluorescent protein (4T1‐EGFP‐N_3_). zCT26 without DBCO arms and 4T1‐EGFP without metabolic labeling were also used as controls. As shown in Figure 2G, substantial zCT26‐DBCO were found to anchor on the surface of 4T1‐EGPF‐N_3_ forming expansive cell‐cell interface after mixture, whereas zCT26 and 4T1‐EGFP distributed separately with minimal contacts. These results validate that zCT26‐DBCO could seize 4T1‐N_3_ via bioorthogonal targeting.

### 2.2 Zombie Cells Induce Pseudo‐Oncolysis in Tumor Cells

We proposed that by anchoring onto tumor cells while emitting DAMPs (e.g., CRT and ATP) around the periphery, “zombie cells” disguise the intact tumor cells as having undergone oncolysis, potentially tricking immune effector cells into engulfment of both zombie and tumor cells (Figure 2H). To test the hypothesis, zCT26 and zCT26‐DBCO with CRT labeling were pre‐stained, tracked by fluorescent 1,1′‐Dioctadecyl‐3,3,3′,3′‐tetramethylindodicarbocyanine, 4‐chlorobenzenesulfonate salt (DiD), and further incubated with 4T1‐EGFP and 4T1‐EGFP‐N_3_, respectively. Flow cytometry scatter plot analysis showed two distinct populations for the mixture of zCT26‐DiD and 4T1‐EGFP, whereas zCT26‐DiD‐DBCO generated considerable clusters with 4T1‐EGFP‐N_3_. Notably, we also detected a substantial increase in CRT fluorescence appearing on EGFP positive cells after the mixture of zCT26‐DiD‐DBCO and 4T1‐EGFP‐N_3_, while the mixture of zCT26‐DiD and 4T1‐EGFP gave rise to a much less CRT signal within EGFP positive cells (Figure 2I). A similar phenomenon was seen for ATP release assessment (Figure S2, Supporting Information).

As a result of their shared display of prophagocytic CRT and ATP signals, substantial phagocytosis of zCT26‐DBCO‐anchored 4T1‐EGFP‐N_3_ by bone‐marrow‐derived dendritic cells (BMDCs) was observed (Figure 2J; Figure S3, Supporting Information). In the control group, where DiD‐labeled BMDCs (BMDCs‐DiD) were added to the premixture of zCT26 and 4T1‐EGFP cells, flow cytometry scatter plot analysis revealed three distinct cell populations initially at 0 hours, representing zCT26, 4T1‐EGFP, and BMDCs‐DiD. After 4 h co‐incubation, phagocytosis of zCT26 cells by BMDCs‐DiD was dominant, while most 4T1‐EGFP cells remained unphagocytosed. In contrast, when zCT26‐DBCO cells were premixed with 4T1‐EGFP‐N_3_ cells followed by addition of stimulated BMDCs‐DiD, flow cytometry analysis revealed two cell populations initially, indicating the anchoring of zCT26‐DBCO onto 4T1‐EGFP‐N_3_ cells through bioorthogonal reaction. At 4 h, only one major cell population was observed, with the majority of 4T1‐EGFP‐N_3_ cells being phagocytosed. These results confirm that the anchoring of zCT26‐DBCO can act as decoys, bridging connections between tumor cells and phagocytic BMDCs.

### 2.3 Construction of Local Delivery Platform

**Figure 3:**
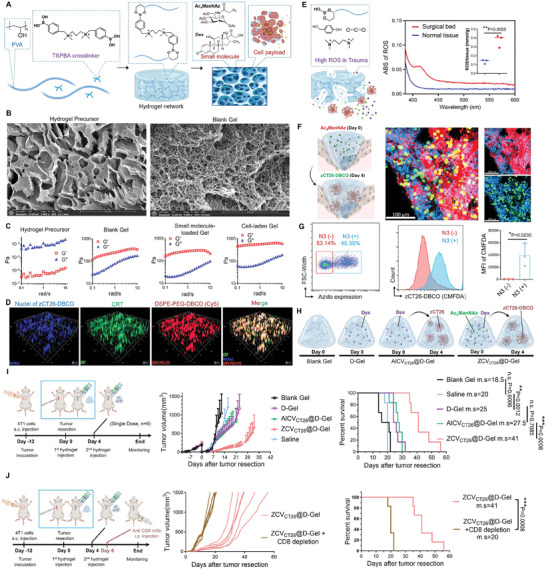
ZCV@D‐Gel inhibits postoperative tumor recurrence. A) Illustration of injectable hydrogel accommodating both small molecules and cells. B) Scanning electron microscopy images of hydrogel precursor solution and blank hydrogel scaffold. Scale bar:10 µm. C) Frequency‐dependent rheological properties of hydrogel precursor, blank hydrogel, small molecule‐loaded hydrogel, and cell‐loaded hydrogel. G': the storage modulus; G””: loss modulus. D) 3D construction images of hydrogel encapsulating zCT26‐DBCO. DSPE‐PEG_2k_‐Cy5 (red) served as fluorescent surrogate for DSPE‐PEG_2k_‐DBCO anchoring on zCT26‐DBCO. CRT (green) and nuclei (blue) were pre‐stained before loading into the hydrogel. E) Quantitative ROS level in surgical bed and normal tissues. F) Fluorescence images of azido expression and zCT26‐DBCO release within the residual tumor tissue, and G) Flow cytometry confirmation of zCT26‐DBCO anchoring on azido‐tagged residual 4T1 tumor cells on day 6 after sequential injection of an Ac_4_ManNAz‐loaded hydrogel on day 0 and a zCT26‐DBCO‐loaded hydrogel on day 4 into the tumor resection cavity. Green, zCT26‐DBCO; Red, azido; Blue, tumor cell nuclei. Scale bar:100 µm. H) Illustration for Blank Gel, D‐Gel, AlCV_CT26_@D‐Gel, or ZCV_CT26_@D‐Gel treatments. I) Tumor regrowth curves and animal survival of post‐surgical tumor models following single dose treatment with saline, Blank Gel, D‐Gel, AlCV_CT26_@D‐Gel, or ZCV_CT26_@D‐Gel (*n* = 6). The arrows indicate the treatment regimens. J) Tumor regrowth curves and animal survival of post‐surgical tumor models over time after cotreatment with ZCV_CT26_@D‐Gel and CD8‐depleting antibodies (*n* = 6). The arrows indicate the treatment regimens. Data are shown as mean ± SD; ^*^ *p* < 0.05, ^**^ *p* <0.01, ^***^ *P* < 0.001, ^****^ *P* < 0.0001.

To effectively deliver “zombie cells” to postoperative tumor sites, an injectable hydrogel was self‐assembled from two syringeable solutions – a hydrogel precursor poly(vinyl alcohol) (PVA) and a crosslinker *N* ^1^‐(4‐boronobenzyl)‐*N* ^3^‐(4‐boronophenyl)‐*N* ^1^,*N* ^1^,*N* ^3^,*N* ^3^‐tetramethylpropane‐1,3‐diaminium (TSPBA).^[^ ^17^ ^]^ The crosslinker contains dual phenylboronic acids that can rapidly react with PVA's diols to form reactive oxygen species (ROS)‐labile pinacol ester bonds, assembling into a scaffold accommodating both small molecules and cells (**Figure** **3**A). Scanning electron microscopy revealed cross‐linked blank hydrogel displayed an organized polymer network texture with abundant pores and channels, distinctly different from the unassembled hydrogel precursor without crosslinking (Figure 3B). Rheological analysis showed the PVA precursor had solution‐like properties before crosslinking. After crosslinking, the storage modulus (G′) exceeded the loss modulus (G′′), confirming sol‐to‐gel transition. This hydrogel property was unaffected by inclusion of small molecules (i.e., Ac_4_ManNAz and dexamethasone) or cell payloads (i.e., zCT26‐DBCO) (Figure 3C). 3D scanning of the cell‐laden hydrogel further showed a uniform dispersion of DAMP‐emitting zCT26‐DBCO within the hydrogel matrix (Figure 3D).

For in vivo application, a dual syringe presented in Figure 1 was used according to a previous protocol.^[^ ^17^ ^]^ PVA aqueous solution or cell‐containing PVA solution was loaded into one syringe, while drug‐containing TSPBA solution or TSPBA aqueous solution was loaded into the other syringe. These solutions were injected directly into tumor resection cavity to form a hydrogel scaffold in situ. In response to surgical trauma where ROS is locally enriched (Figure 3E), the hydrogel can gradually degrade and sustainably release the small molecules and cell payloads in a degradation‐controlled manner.^[^ ^7^, ^11^ ^]^ We also confirmed that immediately after injection into a tumor resection cavity, the hydrogel scaffold could be in situ formed, and completely degraded within 4 days without burst elimination of encapsulated cargos (Figure S4, Supporting Information).

To enable bioorthogonal click anchoring of “zombie cells” to residual tumor cells post‐resection, we performed tumor removal surgery on Balb/c mice with orthotopic 4T1 tumors, intentionally leaving ≈5% of tumor behind. We then sequentially injected two local delivery platforms into the tumor resection cavity: an Ac_4_ManNAz‐loaded hydrogel on day 0 for metabolic glycoengineering and a zCT26‐DBCO‐loaded hydrogel on day 4 for cell‐cell binding. On day 4, prior to the second hydrogel injection, flow cytometry analysis was performed to compare cells isolated from peritumoral normal tissues and residual tumor tissues. The analysis revealed a significantly higher expression of azido in the tumor remnants (Figure S5, Supporting Information). This observation suggests a certain tumor selectivity in azido tagging through metabolic glycoengineering, since tumor cells have higher metabolic demands than normal cells based on previous reports.^[^ ^18^ ^]^ Subsequently after second hydrogel injection, analysis of the tumor tissue section on day 6 showed substantial expression of azido groups and release of zCT26‐DBCO within the residual tumor (Figure 3F). In addition, zCT26‐DBCO fluorescence (pre‐stained with CellTracker probe) appearing in azido‐positive tumor cell subpopulations was markedly higher compared to azido‐negative cell subsets (Figure 3G), validating in vivo selective anchoring of zCT26‐DBCO onto azido‐tagged residual 4T1 tumor cells in this postoperative model.

### 2.4 ZCV@D‐Gel Elicits CD8^+^ T Cell‐Dependent Inhibition of Tumor Recurrence

To investigate the in vivo immunization efficacy of postoperative tumor recurrence inhibition, an orthotopic 4T1 tumor recurrence model after an incomplete tumor resection was established, and randomly divided into four groups. Mice then received a single dose of Blank Gel, D‐Gel, AlCV_CT26_@D‐Gel, or ZCV_CT26_@D‐Gel injected locally in the resection cavity. As illustrated in Figure 3H, Blank Gel referred to hydrogel matrix only; D‐Gel referred to dexamethasone‐loaded hydrogel; AlCV_CT26_@D‐Gel referred to allogeneic cell vaccine strategy in which dexamethasone‐loaded hydrogel was administered on day 0, and CT26 cell corpses succumbing to oncolysis (zCT26)‐loaded hydrogel was administered on day 4; ZCV_CT26_@D‐Gel referred to “zombie cells” vaccine strategy in which dexamethasone and Ac_4_ManNAz co‐loaded hydrogel was administered on day 0, and zCT26‐DBCO‐loaded hydrogel was administered on day 4. As shown in Figure 3I and Figure S6 (Supporting Information), all mice treated with saline or Blank Gel experienced explosive tumor regrowth and died rapidly after tumor resection. Ac_4_ManNAz‐loaded hydrogel exerted no impact on inhibiting the tumor regrowth or extending the survival (Figure S7, Supporting Information). Anti‐inflammatory D‐Gel had marginal effect on controlling tumor or extending survival. By comparison, AlCV_CT26_@D‐Gel failed to further improve the therapeutic outcome. Notably, ZCV_CT26_@D‐Gel significantly retarded the tumor recurrence and extended animal survival post‐surgery. Meanwhile, another group of postsurgical models receiving ZCV_CT26_@D‐Gel were subjected to CD8^+^ T‐cell ablation using CD8‐depleting antibodies. The result showed concurrent depletion of CD8^+^ T cells markedly weakened ZCV@D‐Gel‐mediated tumor regression and compromised survival (Figure 3J), suggesting that ZCV_CT26_@D‐Gel might activate a CD8^+^ T cell immune response to exert 4T1 tumor killing.

Then, biosafety profiles of ZCV_CT26_@D‐Gel were evaluated after its injection into the resection cavity of the postsurgical 4T1 tumor model. Serum chemistry analysis and hematological cell counts showed no significant differences compared to the control group treated with saline (Figure S8, Supporting Information). Histology analysis of major organs (Figure S9, Supporting Information) and host skin tissues surrounding the hydrogel (Figure S10, Supporting Information) revealed no pathological abnormalities or tissue damage. These results indicated no obvious systemic or local toxicity of ZCV_CT26_@D‐Gel.

### 2.5 Bioorthogonal Anchoring and Oncolysis Immunization are Dispensable for ZCV@D‐Gel Efficacy

**Figure 4:**
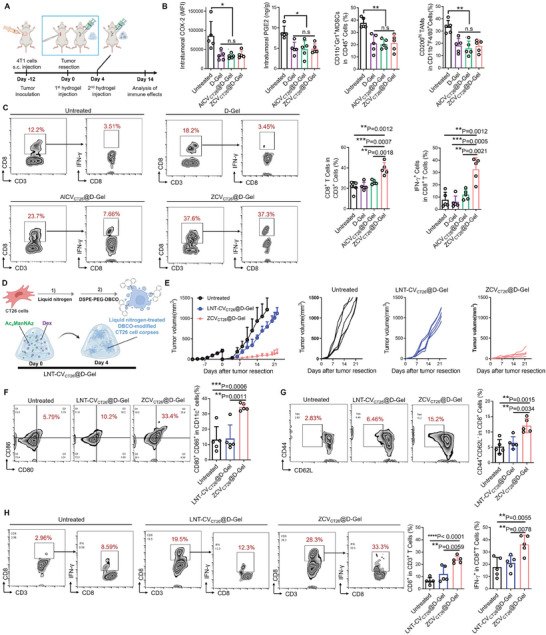
ZCV@D‐Gel activates CD8^+^ T cell response. A) Schedules for treatment regimen. B) Measurement of inflammatory factors (PGE2 and COX‐2), and immunosuppressive cells (CD11b^+^Gr1^+^ MDSCs and CD206^hi^CD11b^+^F4/80^+^ TAMs), and C) analysis of tumor‐infiltrating CD3^+^CD8^+^ T cells and CD8^+^IFN‐γ^+^ T cells in the residual tumors following the postsurgical interventions with D‐Gel, AlCV_CT26_@D‐Gel, or ZCV_CT26_@D‐Gel (*n* = 5). D) Illustration for LNT‐CV_CT26_@D‐Gel. E) Average and individual growth curves of recurrent tumors post‐surgery, frequencies of F) CD80^+^CD86^+^CD11c^+^ mature DCs in tumor‐draining lymph node, G) splenic CD44^+^CD62L^−^ memory effector cells (gated on CD8^+^ T cells), and H) CD8^+^IFN‐γ^+^ tumor‐reactive T cells in residual tumors after postsurgical interventions with LNT‐CV_CT26_@D‐Gel and ZCV_CT26_@D‐Gel (*n* = 5). Data are shown as mean ± SD; ^*^ *p* < 0.05, ^**^ *p* <0.01, ^***^ *P* < 0.001, ^****^ *P* < 0.0001.

We next analyzed the immune status of residual tumors following the above treatments (**Figure** **4**A). After injection into the resection cavity, postsurgical intervention with D‐Gel, AlCV_CT26_@D‐Gel, and ZCV_CT26_@D‐Gel all decreased the local levels of pro‐inflammatory cytokines cyclooxygenase‐2 (COX‐2) and prostaglandin E2 (PGE2), owing to the effect of dexamethasone. This further led to a significant reduction in the frequencies of myeloid‐derived suppressor cells (MDSCs) and tumor‐associated macrophages (TAMs) in the tumor remnants (Figure 4B). This aligned with previous finding that alleviating surgery‐induced inflammation could neutralize its effect on exacerbating immunosuppression.^[^ ^7^ ^]^ Although D‐Gel significantly shaped the residual tumor landscape, potentially unleashing pre‐existing T cells, it failed to mobilize more T cells to the tumors. Similarly, AlCV_CT26_@D‐Gel did not promote tumor infiltration of CD8^+^ T cells, likely due to the known fact that allogeneic cell‐derived vaccines lack patient‐specific neoantigen reactivity, which is thought to be important for generating anti‐tumor immunity. Analogous to installing DAMP‐producing factories on tumor cells, ZCV_CT26_@D‐Gel with cell anchoring capability can act as an extension of the tumor cells to increase overall immunogenicity and boost in situ vaccination effects. Consequently, ZCV_CT26_@D‐Gel recruited a significantly increased number of CD8^+^ T cells to the tumor site with an elevated proportion secreting IFN‐γ to exert tumoricidal activity (Figure 4C).

In another individual experiment, a ZCV_CT26_@D‐Gel analogue (LNT‐CV_CT26_@D‐Gel) was designed that retained the bioorthogonal anchoring function but lacked the self‐adjuvanting properties. LNT‐CV_CT26_@D‐Gel involved administering a dexamethasone and Ac_4_ManNAz co‐loaded hydrogel on day 0, followed by a hydrogel loaded with liquid nitrogen‐treated DBCO‐modified CT26 cell corpses on day 4 (Figure 4D). In accordance with previous studies,^[^ ^19^, ^20^ ^]^ liquid nitrogen treatment could abrogate cancer cells’ proliferation ability and pathogenicity, but still maintained low immunogenicity (Figure S11, Supporting Information). Following the same treatment regimen, ZCV_CT26_@D‐Gel generated a reproducible outcome with significant inhibition of recurrent tumors in postsurgical mice, whereas LNT‐CV_CT26_@D‐Gel exerted moderate anti‐tumor effect (Figure 4E). Immune analyses at the endpoint showed that ZCV_CT26_@D‐Gel, with its capability to induce pseudo‐oncolysis in tumor cells upon bioorthogonal anchoring, resulted in higher frequencies of CD80^+^CD86^+^CD11c^+^ mature DCs in tumor‐draining lymph nodes (TDLNs) (Figure 4F). Moreover, compared with untreated control, ZCV_CT26_@D‐Gel dramatically increased the frequency of CD11c^+^CD11b^+^Ly6c^+^ DCs in the TDLNs, a crucial subset of antigen‐presenting cells (APCs) that played significant roles in the presentation of tumor antigens.^[^ ^21^ ^]^ These APCs also up‐regulated the level co‐stimulatory marker CD86 (Figure S12, Supporting Information). In comparison, despite having bioorthogonal anchoring capability as well, the impact of poorly immunogenic LNT‐CV_CT26_@D‐Gel was negligible. Similar trends were also observed in the frequencies of CD44^+^CD62L^−^ memory effector CD8^+^ T cells in spleen (Figure 4G), and CD8^+^IFN‐γ^+^ tumor‐reactive T cells in residual tumors (Figure 4H).

These results highlighted bioorthogonal anchoring and oncolysis immunization as two dispensable functionalities enabling ZCV_CT26_@D‐Gel to “seize” and “assimilate” the heterogeneous residual tumors to potently stimulate a tumor‐specific immune response.

### 2.6 Allogeneic ZCV_CT26_@D‐Gel Establishes Adaptive Immunity against 4T1 Tumor Re‐Challenge

**Figure 5:**
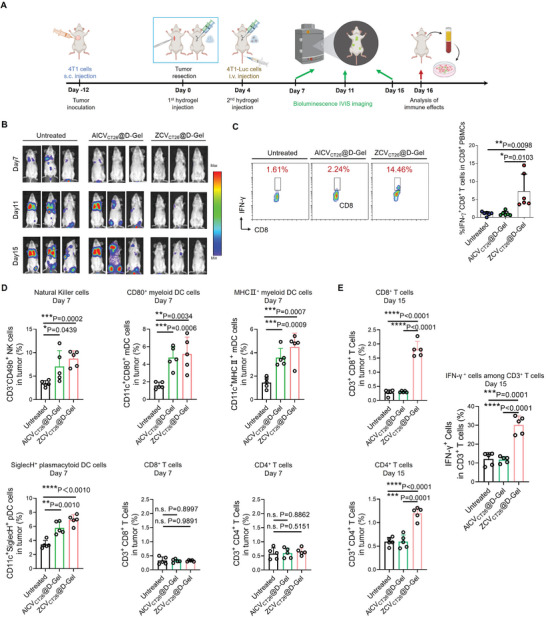
Allogeneic ZCV_CT26_@D‐Gel establishes systemic immune response against disseminated 4T1 tumor re‐challenge. A) Schematic illustration of treatment schedule and tumor re‐challenge. B) Bioluminescent images of disseminated 4T1‐Luc cells in mice (*n* = 3). C) Flow cytometry scatter plot representation and quantification analysis of CD8^+^IFN‐γ^+^ T cell percentage among PBMCs upon coculturing with 4T1 cells (gated on CD8^+^ cells, *n* = 6). PBMCs were isolated from the mice that received tumor resection, postoperative interventions with AlCV^CT26^@D‐Gel or ZCV^CT26^@D‐Gel, and tumor re‐challenge. D) Flow cytometry analysis of natural killer cells (CD3^−^CD49b^+^), plasmacytoid dendritic cells (CD11c^+^SiglecH^+^), myeloid dendritic cells (CD11c^+^CD80^+^, CD11c^+^MHCII^+^), and CD3^+^ T cells (CD8^+^, CD4^+^) in residual tumors on day 7. E) Flow cytometry analysis of CD3^+^ T cells (CD8^+^, CD4^+^) and IFN‐γ^+^ cells among CD3^+^ T cells in residual tumors on day 15. Data are shown as mean ± SD; ^*^ *p* < 0.05, ^**^ *p* <0.01, ^***^ *P* < 0.001, ^****^ *P* < 0.0001.

To investigate whether ZCV_CT26_@D‐Gel induced adaptive immunity against 4T1 tumors, orthotopic 4T1 tumor‐bearing mice that received tumor resection and postoperative interventions with ZCV_CT26_@D‐Gel were intravenously re‐challenged with luciferase‐expressing 4T1 (4T1‐Luc) cells followed by bioluminescence imaging until immune analysis at the endpoint (**Figure** **5**A). Extensive growth of 4T1‐Luc cells was observed in untreated post‐surgical recurrence models or those treated with AlCV_CT26_@D‐Gel. In contrast, ZCV_CT26_@D‐Gel‐treated mice were largely resistant to 4T1‐Luc cell re‐challenge (Figure 5B). Moreover, coculturing peripheral blood mononuclear cells (PBMCs) isolated from ZCV_CT26_@D‐Gel treated mice with live 4T1 cells significantly expanded frequencies of tumor cell‐reactive CD8^+^IFN‐γ^+^ T cells, whereas PBMCs from other groups failed to generate the 4T1 cell‐specific response (Figure 5C). These results indicate that local delivery of ZCV_CT26_@D‐Gel to 4T1 tumor residuals in resection cavity induced systemic T cell memory response and showed abscopal anti‐tumor effect against disseminated 4T1 tumor re‐challenge.

We conducted further analysis of the immune status in the residual tumors of re‐challenged mice on day 7 and day 15. Day 7, three days after vaccination, is a period of assessing immediate innate immune response.^[^ ^22^ ^]^ As shown in Figure 5D, we observed an increase in the number of natural killer cells (CD3^−^CD49b^+^), plasmacytoid dendritic cells (CD11c^+^SiglecH^+^), and myeloid dendritic cells (CD11c^+^CD80^+^, CD11c^+^MHCII^+^) in residual tumors of both AlCV_CT26_@D‐Gel and ZCV_CT26_@D‐Gel groups. This could be the result of vaccination‐induced systemic inflammation that activated immediate innate immunity.^[^ ^23^ ^]^ However, we did not observe any significant change in the frequencies of CD8^+^ or CD4^+^ T cell (Figure 5D), indicating that adaptive immunity had not yet been established in these groups. In contrast, on day 15, while AlCV_CT26_@D‐Gel still exerted no impact on T cell activation due to low antigenic relevance of CT26 cell‐derived vaccines to 4T1 tumors,^[^ ^7^, ^8^, ^14^ ^]^ ZCV_CT26_@D‐Gel significantly expanded the frequencies of tumor‐infiltrating CD8^+^CD3^+^ T cells, CD4^+^CD3^+^ T, and IFN‐γ‐secreting T cells (Figure 5E).

Collectively, we demonstrated the following: 1) initially on day 7, immediate innate immune responses by both AlCV_CT26_@D‐Gel and ZCV_CT26_@D‐Gel vaccination were dominate, resulting in greater inhibition of disseminated 4T1‐Luc cells metastasis to the lungs than the untreated control groups; 2) as time progressed to day 15, innate immune response alone was inadequate to suppress the development and pulmonary metastasis of 4T1‐Luc cells in AlCV_CT26_@D‐Gel‐treated group, whereas 4T1‐specific adaptive immunity has been eventually established for ZCV_CT26_@D‐Gel‐treated group leading to remarkable resistance against 4T1‐Luc cell growth.

### 2.7 Off‐The‐Shelf Products of Allogeneic “Zombie” Vaccines Evenly Match with Autologous Vaccine

Encouraged by the successful ZCV_CT26_@D‐Gel elicitation of immune response against residual 4T1 breast tumors post‐surgery, we next investigated the general applicability of the “zombie” technique from other allogeneic cell sources, namely B16 melanoma cells and LLC‐1 Lewis lung cancer cells. Following an identical procedure, ZCV_B16_@D‐Gel/ ZCV_LLC‐1_@D‐Gel involved administering a dexamethasone plus Ac_4_ManNAz co‐loaded hydrogel on day 0, followed by a hydrogel loaded with DBCO‐modified B16/LLC‐1 cell corpses succumbing to oncolysis on day 4. As conventional controls, AlCV_B16_@D‐Gel/ AlCV_LLC‐1_@D‐Gel involved administrating a dexamethasone loaded hydrogel on day 0, followed by a hydrogel loaded with B16/LLC‐1 cell corpses succumbing to oncolysis on day 4. In addition, after incomplete resection of orthotopic 4T1 breast tumors, we further compared the anticancer performances of these allogeneic vaccines to the autologous vaccine designated AuCV_4T1_@D‐Gel, which involved administrating a dexamethasone‐loaded hydrogel on day 0, followed by a hydrogel loaded with 4T1 cell corpses succumbing to oncolysis on day 4.

**Figure 6:**
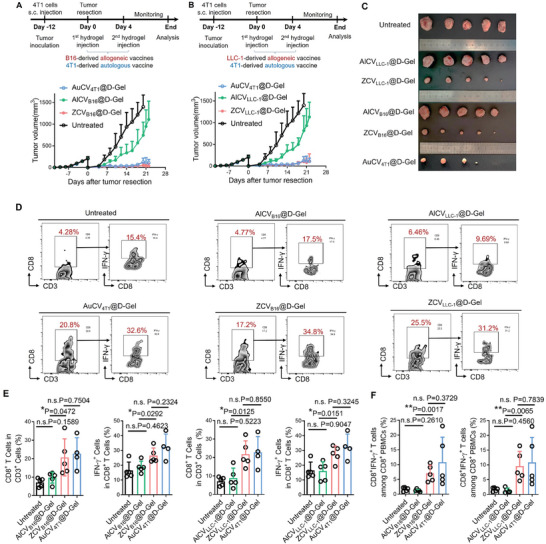
Allogeneic ZCV@D‐Gel shows comparable efficacy with autologous cell vaccine. A) Growth curves of recurrent 4T1 breast tumors post‐surgery, following single dose treatment with melanoma B16 cell‐derived allogeneic vaccine (ZCV_B16_@D‐Gel, AlCV_B16_@D‐Gel), or 4T1‐derived autologous vaccine (AuCV_4T1_@D‐Gel). *n* = 5. The arrows indicate the treatment regimens. (B) Growth curves of recurrent 4T1 breast tumors post‐surgery, following single dose treatment with lung carcinoma LLC‐1 cell‐derived allogeneic vaccine (ZCV_LLC‐1_@D‐Gel, AlCV_LLC‐1_@D‐Gel), or 4T1‐derived autologous vaccine (AuCV_4T1_@D‐Gel). *n* = 5. The arrows indicate the treatment regimens. C) Photographs of orthotopic 4T1 tumors collected at the endpoint after indicated treatments. D) Flow cytometry scatter plot representation and E) quantified analysis of CD8^+^IFN‐γ^+^ tumor‐reactive T cells in residual tumors after indicated postsurgical interventions (*n* = 5). F) Flow cytometry analysis of CD8^+^IFN‐γ^+^ T cell percentage among PBMCs upon coculturing with 4T1 cells (gated on CD8^+^ cells, *n* = 5). PBMCs were isolated from the mice that received tumor resection and indicated postoperative interventions. Data are shown as mean ± SD; ^*^ *p* < 0.05, ^**^ *p* <0.01, ^***^ *P* < 0.001, ^****^ *P* < 0.0001.

As shown in **Figure** **6**A–C and Figure S13 (Supporting Information), postsurgical intervention with AlCV_B16_@D‐Gel and AlCV_LLC‐1_@D‐Gel generated unsatisfactory outcome with rapid growth of recurrent 4T1 tumors. Notably, despite using allogeneic cell sources, “zombie” vaccines ZCV_B16_@D‐Gel and ZCV_LLC‐1_@D‐Gel effectively controlled the growth of tumor remnants, with the anti‐tumor capabilities evenly matched with AuCV_4T1_@D‐Gel. In similar trends, AlCV_B16_@D‐Gel and AlCV_LLC‐1_@D‐Gel failed to promote T cell infiltration into residual tumors compared to untreated control. In contrast, ZCV_B16_@D‐Gel and ZCV_LLC‐1_@D‐Gel resulted in a significant increase in the frequency of intratumoral CD8^+^IFN‐γ^+^ T cells, showing immunostimulation comparable to AuCV_4T1_@D‐Gel (Figure 6D,E). Furthermore, coculturing PBMCs from post‐surgical mice with live 4T1 cells dramatically expanded reactive CD8^+^IFN‐γ^+^ T cells for ZCV_B16_@D‐Gel, ZCV_LLC‐1_@D‐Gel, and AuCV_4T1_@D‐Gel treated groups, whereas AlCV_B16_@D‐Gel or AlCV_LLC‐1_@D‐Gel did not increase IFN‐γ^+^CD8^+^ T cells beyond basal levels (Figure 6F). This indicates “zombie” vaccines, via pseudo‐oncolysis of “seized” tumor cells, can establish immune responses specific to allogeneic targets.

### 2.8 Repetitive Stimulation by ZCV@D‐Gel as a Generalizable Framework to Produce Long‐Term Effect

Given the low antigenic relevance between 4T1 breast cancer cells and CT26 colon tumors,^[^ ^7^, ^8^, ^14^ ^]^ we then assessed the generalizability of ZCV@D‐Gel platform by evaluating the therapeutic efficacies of (i) ZCV_4T1_@D‐Gel that delivered 4T1 cell‐derived “zombie cells” in treating subcutaneous CT26 tumor recurrence models, and (ii) ZCV_CT26_@D‐Gel that delivered CT26 cell‐derived “zombie cells” in treating orthotopic 4T1 tumor recurrence model.

**Figure 7:**
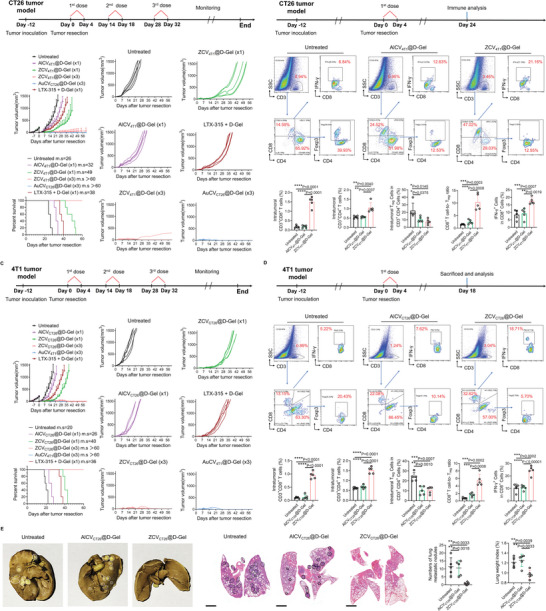
Repetitive ZCV@D‐Gel stimulation generates long‐term effect in different postsurgical tumor models. A) Tumor regrowth curves and animal survival of recurrent CT26 colon tumors post‐surgery, following a single dose of AlCV_4T1_@D‐Gel (×1) as allogeneic cell vaccine, a single dose of ZCV_4T1_@D‐Gel (×1) as “zombie cell” vaccine, a single dose of LTX‐315+D‐Gel as in situ vaccine, biweekly three‐cycle of ZCV_4T1_@D‐Gel (×3), and biweekly three‐cycle of AuCV_CT26_@D‐Gel (×3) as autologous cell vaccine. The arrows indicate the treatment regimens. B) Flow cytometry analysis of immune T cells, including CD3^+^CD8^+^ T cells, CD3^+^CD4^+^ T cells, Foxp3^+^CD4^+^ Tregs, CD8^+^ T cells to Tregs ratio, and IFN‐γ^+^ cells among CD8^+^ T cells, in residual CT26 tumors following a single dose of AlCV_4T1_@D‐Gel and ZCV_4T1_@D‐Gel on day 24, the endpoint of postsurgical mice left untreated. C) Tumor regrowth curves and animal survival of recurrent 4T1 breast tumors post‐surgery, following a single dose of AlCV_CT26_@D‐Gel (×1) as allogeneic cell vaccine, a single dose of ZCV_CT26_@D‐Gel (×1) as “zombie cell” vaccine, a single dose of LTX‐315+D‐Gel as in situ vaccine, biweekly three‐cycle of ZCV_CT26_@D‐Gel (×3), and biweekly three‐cycle of AuCV_4T1_@D‐Gel (×3) as autologous cell vaccine. The arrows indicate the treatment regimens. D) Flow cytometry analysis of immune T cells, including CD3^+^CD8^+^ T cells, CD3^+^CD4^+^ T cells, Foxp3^+^CD4^+^ Tregs, CD8^+^ T cells to Tregs ratio, and IFN‐γ^+^ cells among CD8^+^ T cells, in residual 4T1 tumors following a single dose of AlCV_CT26_@D‐Gel and ZCV_CT26_@D‐Gel on day 24, the endpoint of postsurgical mice left untreated. E) Pulmonary metastasis evaluation in postsurgical 4T1 tumor models receiving a single dose of AlCV_CT26_@D‐Gel and ZCV_CT26_@D‐Gel on day 24, the endpoint of postsurgical mice left untreated. Scale bar, 2000 mm. *N* = 5. Data are shown as mean ± SD; ^*^ *p* < 0.05, ^**^ *p* <0.01, ^***^ *P* < 0.001, ^****^ *P* < 0.0001.

Mice bearing CT26 tumors and receiving incomplete tumor resection were randomly divided and given the following treatments: left untreated, a single dose of AlCV_4T1_@D‐Gel (×1) as allogeneic cell vaccine, a single dose of ZCV_4T1_@D‐Gel (×1) as “zombie cell” vaccine, a single dose of LTX‐315+D‐Gel as in situ vaccine, biweekly three‐cycle of ZCV_4T1_@D‐Gel (×3), and biweekly three‐cycle of AuCV_CT26_@D‐Gel (×3) as autologous cell vaccine. As shown in **Figure** **7**A, compared to untreated mice, post‐surgical intervention with AlCV_4T1_@D‐Gel (×1) had limited efficacy in inhibiting CT26 tumor regrowth or extending survival, due to low antigenic relevance of 4T1 cell‐derived vaccines to CT26 tumors. On the other hand, ZCV_4T1_@D‐Gel (×1) significantly slowed down CT26 tumor regrowth and prolonged survival after surgery. Analysis of immune T cells following single‐dose treatment revealed that AlCV_4T1_@D‐Gel (×1) failed to promote CD8^+^ T cell infiltration in residual CT26 tumors, while ZCV_4T1_@D‐Gel (×1) successfully activated CD8^+^ T cell response (Figure 7B). Moreover, ZCV_4T1_@D‐Gel (×1) demonstrated superior antitumor efficacy compared to LTX‐315+D‐Gel, likely due to challenges in directly injecting LTX‐315 into microscopic and scattered tumor remnants, which are difficult to access for effective delivery. Although a single dose of ZCV_4T1_@D‐Gel induced an antitumor immune response, it did not have a long‐term effect. ZCV_4T1_@D‐Gel (×1)‐treated residual CT26 tumors eventually grew large, leading to the death of all mice. To address this limitation, repetitive immune stimulation using ZCV_4T1_@D‐Gel was proposed. Remarkably, ZCV_4T1_@D‐Gel (×3) effectively suppressed CT26 tumor recurrence post‐surgery and demonstrated a long‐term effect. The treated mice exhibited relatively small tumor volumes and survived for over two months (Figure 7A). Furthermore, ZCV_4T1_@D‐Gel (×3) showed comparable therapeutic outcomes to AuCV_CT26_@D‐Gel (×3), suggesting that off‐the‐shelf products of allogeneic “zombie” vaccines can be as effective as autologous vaccines.

Similar results regarding antitumor efficacy, animal survivorship, and immune stimulation were observed in postsurgical model of orthotopic 4T1 tumors comparing ZCV_CT26_@D‐Gel (×1) and AlCV_CT26_@D‐Gel (×1), and comparing ZCV_CT26_@D‐Gel (×3) and AuCV_4T1_@D‐Gel (×3). Briefly, AlCV_CT26_@D‐Gel (×1) exhibited limited effectiveness due to low antigenic relevance, whereas ZCV_CT26_@D‐Gel (×1) outperformed AlCV_CT26_@D‐Gel (×1) in retarding the regrowth of post‐operative 4T1 tumors. Additionally, LTX‐315+D‐Gel exerted inferior antitumor efficacy compared to ZCV_CT26_@D‐Gel (×1), due to challenges in delivering LTX‐315 to scattered tumor remnants. Still, long‐term inhibition of 4T1 tumor residuals could not be achieved by ZCV_CT26_@D‐Gel (×1) with only one single dose, which may require multiple doses for continuous activation and reinforcement of antitumor immune response over an extended period. Notably, through repetitive stimulation, ZCV_CT26_@D‐Gel (×3) eradicated the recurrent 4T1 tumor post‐surgery and achieved a long‐term 100% survival rate, showing comparable outcomes to AuCV_4T1_@D‐Gel (×3) (Figure 7C).

Further deciphering of T cells (Figure 7D) revealed that ZCV_CT26_@D‐Gel resulted in a significantly higher recruitment of CD3^+^ T cells to residual 4T1 tumor compared to the untreated control group, 18 days after surgery. There was an approximately ninefold increase in CD3^+^CD8^+^ T cells and a 2.5‐fold increase in CD3^+^CD4^+^ T cells, both of which are beneficial for antitumor immunity. Although AlCV_CT26_@D‐Gel failed to promote tumor infiltration of CD3^+^ T cells, it led to a similar reduction in Foxp^+^CD4^+^ regulatory T cell (Tregs) within CD4^+^ T cell population as ZCV_CT26_@D‐Gel. This could be ascribed to the effect of D‐Gel, since inflammation alleviation in tumor microenvironment has been reported to deplete Tregs.^[^ ^24^ ^]^ Consequently, with the concomitant increase in tumor‐infiltrating CD8^+^ T cells and decrease in Tregs, ZCV_CT26_@D‐Gel substantially elevated CD8^+^ T cells to Tregs ratio, leading to significant expansion of tumor cell‐reactive T cells (IFN‐γ^+^CD8^+^), suggesting an enhanced antitumor immune response.

Considering orthotopic 4T1 breast tumors have a high propensity for spontaneous metastasis, particularly to distant lung through hematogenous dissemination,^[^ ^7^, ^11^ ^]^ we proceed to assess the effectiveness of ZCV_CT26_@D‐Gel in preventing metastasis. The evaluation was performed 18 days after surgery, the endpoint for control mice that underwent tumor resection but received no further treatment when their residual primary tumor reached a volume exceeding 1500 mm^3^. Notably, untreated mice and those treated with AlCV_CT26_@D‐Gel exhibited visible tumor nodules in the lung, indicating the presence of lung metastasis. In contrast, ZCV_CT26_@D‐Gel‐treated mice showed minimal evidence of metastatic foci in the lung (Figure 7E). This outcome can be attributed to a significant reduction in primary tumor burden (Figure 7C) and the establishment of systemic antitumor memory, as previously described (Figure 5).

Collectively, the effectiveness of ZCV_4T1_@D‐Gel on postsurgical model of CT26 tumors, and ZCV_CT26_@D‐Gel on postsurgical model of 4T1 tumors demonstrated ZCV@D‐Gel platform as a generalizable framework.

## 3 Discussion

In the present study, we designed a “zombie cell” vaccine to stimulate immune responses specific to residual tumors after surgery. “Zombie cells” were referred to allogeneic cell corpses that were pretreated with clinical‐grade oncolytic peptide of LTX‐315, succumbing to oncolysis, emitting adjuvanting DAMPs, further armed with artificial ligand, and fabricated ex vivo (Figure 2A,B). Akin to zombie behaviors of the walking dead preying on the living and assimilating the heterogeneous, these cell corpses were able to seize tumor cells (Figure 2E–G) and spread immunostimulatory properties (Figure 2H,I), resulting in pseudo‐oncolysis of tumor cells to convert them into immunogenic targets for enhanced immune phagocytosis (Figure 2J). For postsurgical delivery, we further constructed a ZCV@D‐Gel delivery platform involving stepwise in situ gelation in resection cavity. A tumor‐priming hydrogel first alleviated local inflammation to reverse immunosuppression (Figure 4B) while metabolically glycoengineering residual tumor cells with artificial receptors (Figure 3F; Figure S5, Supporting Information). The subsequent immune‐activating hydrogel then delivered premade “zombie cells” for bioorthogonal cell‐cell conjugation (Figure 3G). Compared to AlCV@D‐Gel without “seizing” capability and LNT‐CV@D‐Gel without “assimilating” capability, ZCV@D‐Gel with both functionalities could more effectively mature DCs, mobilize T cells to infiltrate and combat residual tumors (Figure 4). Despite using allogeneic cell sources, ZCV@D‐Gel still established specific immune memory against primary tumor re‐challenge, conferring much less susceptibility to tumor immune evasion (Figure 5). Markedly, allogeneic ZCV@D‐Gel showed comparable therapeutic effect with autologous AuCV@D‐Gel on stimulating tumor‐specific immunity (Figures 6 and 7).

Regarding the advantages over direct intratumoral injection of LTX‐315, ZCV@D‐Gel offers the potential to overcome resistance mechanisms associated with LTX‐315 and address the challenges in delivering LTX‐315 intratumorally after surgery. LTX‐315, a non‐viral peptide resembling oncolytic viruses in their cationic and amphipathic nature, is under clinical development for solid tumor treatment.^[^ ^25^ ^]^ Its therapeutic mechanism involves perturbing cancer cell membranes and inducing ICD.^[^ ^26^ ^]^ However, tumor heterogeneity, characterized by diverse genetic profiles, can give rise to resistant subpopulations that modify plasma membrane components (e.g., anionic glycan), limiting the effectiveness of oncolytic peptides.^[^ ^27^ ^]^ Additionally, various various immunosuppressive mechanisms hinder the effective induction of ICD, including intratumoral bacteria modulation,^[^ ^28^ ^]^ compensatory autophagy stimulation,^[^ ^29^ ^]^ STING pathways dysregulation,^[^ ^30^ ^]^ anti‐apoptotic proteins overexpression,^[^ ^31^ ^]^ and DNA damage repair pathway activation.^[^ ^32^ ^]^ These barriers ultimately result in the defective release of DAMPs that function as danger signals necessary for alerting the antitumor immune system. In our design, we go beyond intratumoral injection of LTX‐315 to convert tumors into in situ vaccines. Instead, we take one step ahead by locally delivering allogeneic “zombie cells” succumbing to oncolysis, serving as an antitumor vaccine. These premade cell corpses are fabricated ex vivo by treating allogeneic cells with LTX‐315 and conferring them with a bioorthogonal property. We specifically choose cell lines susceptible to LTX‐315, ensuring that the “zombie cells” release a substantial amount of adjuvanting DAMPs, boosting their immunogenicity. Thus, this strategy may circumvent resistance mechanisms that hinder the effectiveness of LTX‐315. Currently, administration of LTX‐315 is subject to intratumoral injection.^[^ ^25^ ^]^ This poses a significant challenge for post‐operative intervention. Residual tumors that may be present at surgical margins are typically microscopic, scattered sporadically in adjacent tissues, and often inaccessible for adequate delivery of the oncolytic peptide. In this study, the delivery strategy involves stepwise gelation within the tumor resection cavity: a tumor‐priming hydrogel reduces inflammation and glycoengineer tumor cells with receptor‐like azido groups, followed by an immunocativatory hydrogel carrying DBCO‐modified “zombie cells” that target azido‐tagged tumor cells via bioorthogonal conjugation and stimulate anti‐tumor immunity. Since tumor cells have higher metabolic demands than normal cells based on previous reports,^[^ ^18^ ^]^ we postulated a certain tumor selectivity in azido tagging through metabolic glycoengineering. Indeed, we observed selective azido tagging of tumor cells after implantation of Ac_4_ManNAz‐loaded hydrogel in tumor resection cavity, resulting in higher surface azido group expression in residual tumor tissues compared to normal peritumoral tissues (Figure S5, Supporting Information). Sequentially, the second hydrogel successfully anchored “zombie cells” to azido‐tagged cells through bioorthogonal reaction‐mediated targeting (Figure 3F,G). Moreover, we further showed that ZCV@D‐Gel exhibited superior therapeutic efficacy in postsurgical tumor models compared to concurrent treatment with LTX‐315+D‐Gel (Figure 7A,C). In the LTX‐315+D‐Gel group, only visible tumor remnants were treated by intratumoral injection of LTX‐315, followed by immediate implantation of D‐Gel.

Regarding the comparison with conventional cancer vaccines, ZCV@D‐Gel is an allogeneic cell vaccine formulation that combines both off‐the‐shelf and personalized characteristics. Existing cancer vaccines can be categorized as predefined shared antigen cancer vaccines or personalized vaccines.^[^ ^1^, ^2^, ^3^, ^4^, ^5^ ^]^ Predefined shared antigen vaccines target common antigens found in cancers and offer the advantage of being “off‐the‐shelf” for timely use. However, their effectiveness may be limited by antigen heterogeneity across patients and tumor types. Personalized vaccines, on the other hand, are tailored to an individual's tumor characteristics, reducing susceptibility to tumor immune evasion. However, they are not readily available and require time‐consuming processes such as sequencing and preparation of patient‐derived neoantigens, which may not align with the narrow window for post‐surgical tumor intervention. For instance, autologous cell vaccines from patient's own tumor cells possess full repertoire of potential antigens, but they are often plagued with time‐consuming acquisition and genetic modification to boost their immunogenicity.^[^ ^3^, ^4^ ^]^ In this study, allogeneic “zombie cell” vaccine is considered “off‐the‐shelf” products because they typically use cancer cell lines that have been adapted for growth and engineering in culture, and can be manufactured in batches ahead of time and don't rely on materials derived individually from each patient, which makes them readily available. Additionally, these “zombie cells” are capable of assimilating heterogeneous tumor by seizing cancer cells and triggering pseudo‐oncolysis effect by spreading adjuvant infection, turning tumor residuals into immunogenic targets, and successfully mobilizing specific T cell response. This innovative strategy combines the benefits of predefined shared antigen cancer vaccines and personalized vaccines: ZCV@D‐Gel is an “off‐the‐shelf” product conductive to timely treatment of residual tumors post‐surgery, while also a “personalized” product overcoming the limited antigenic relevance of allogeneic vaccines to a patient's heterogenous tumor.

Despite these advantages, there are certain limitations that need to be addressed for the further development of ZCV@D‐Gel. One limitation is the insufficient presentation of tumor‐associated antigens and tumor‐specific antigens due to the resection of the majority of tumor tissues during surgery. In clinics, debulking surgery is intentionally incomplete tumor resection due to tumor size, location, stage, patient comorbidities, technical challenges, preservation of organ function.^[^ ^33^ ^]^ As a result, multimodality therapy is required to address residual tumor. The goal of debulking surgery is to reduce the tumor burden and improve the effectiveness of subsequent therapies. Therefore, ZCV@D‐Gel can be applied under circumstances where tumor debulking is performed for neoadjuvant therapeutic intent so that ZCV@D‐Gel can have a greater pool of antigens for generating antigen‐specific T cell responses. Another limitation lies in the sequential gelation process for a tumor‐priming hydrogel and an immunocativatory hydrogel in the tumor resection cavity within a short timeframe. To address this, there is a clear need for a simplified delivery approach that can 1) co‐load multiple payloads in an “all‐in‐one” fashion and 2) sequentially release them in a spatiotemporally regulated manner. As a potential solution in the future study, we might consider the use of binary hydrogels,^[^ ^34^, ^35^ ^]^ with an outer layer initiatively releasing dexamethasone and Ac_4_ManNAz while an inner layer accommodating pre‐made “zombie cells”, enabling an all‐in‐one time‐programmed sequential delivery.

## 4 Conclusion

In summary, we have designed a stepwise gelation platform to deliver “zombie cells” to tumor resection cavity. These “zombie cells” are capable of assimilating heterogeneous tumor by seizing cancer cells and triggering pseudo‐oncolysis effect by spreading adjuvant infection, turning tumor residuals into immunogenic targets and successfully mobilizing specific T cell response. This strategy shows the potential of combining the benefits of off‐the‐shelf availability and personalized relevance to a patient's heterogenous antigens, which suggests an alternative strategy for timely therapy after tumor surgery.

## Conflict of Interest

The authors declare no conflict of interest.

## Supporting information

Supporting Information

## Data Availability

The data that support the findings of this study are available from the corresponding author upon reasonable request.
