# Supplementary material for: Allogeneic “Zombie Cell” as Off‐The‐Shelf Vaccine for Postsurgical Cancer Immunotherapy
Source: Adv Sci (Weinh). 2024 Jan 26;11(13):2307030. doi: 10.1002/advs.202307030 (PMC10987105; doi:10.1002/advs.202307030)
Supplement: Supplementary file 1 — Supporting Information [file ADVS-11-2307030-s001.pdf]

ADVANCED SCIENCE
Open Access

Supporting Information

for *Adv. Sci.*, DOI 10.1002/advs.202307030

Allogeneic "Zombie Cell" as Off-The-Shelf Vaccine for Postsurgical Cancer Immunotherapy

*Bo Li, Ping Zhang, Junlin Li, Rui Zhou, Minglu Zhou, Chendong Liu, Xi Liu, Liqiang Chen and Lian Li**

Supporting Information

Allogeneic “Zombie Cell” as Off-the-Shelf Vaccine for Post-Surgical Cancer Immunotherapy

Bo Li, Ping Zhang, Junlin Li, Rui Zhou, Minglu Zhou, Chendong Liu, Xi Liu, Liqiang Chen, Lian Li*

B. Li, P. Zhang, J. Li, Dr. M. Zhou, C. Liu, X. Liu, Dr. L. Chen and Prof. L. Li
Key Laboratory of Drug-Targeting and Drug Delivery System of the Education Ministry and Sichuan Province, Sichuan Engineering Laboratory for Plant-Sourced Drug and Sichuan Research Center for Drug Precision Industrial Technology, West China School of Pharmacy, Sichuan University
Chengdu 610041, China.
E-mail: liliantripple@163.com

Dr. R. Zhou
NMPA Key Laboratory for Technical Research on Drug Products In Vitro and In Vivo Correlation, Sichuan Institute for Drug Control
Chengdu 611730, China

SUPPLEMENTAL MATERIALS AND METHODS

Cell lines and animals

4T1 murine breast cancer cells, luciferase expressing 4T1 cells (4T1-Luc), LLC-1 murine Lewis lung cancer cells, B16F10 murine melanoma cells and CT26 murine colorectal cancer cells were purchased from Chinese Academy of Science Cell bank (Shanghai, China). 4T1 cells transfected with enhanced green fluorescent protein (4T1-EGFP) were purchased from Yuan Jin Biotechnology Company (Guangzhou, China). Furthermore, 4T1, 4T1-EGFP, 4T1-Luc, CT26, and B16F10 cell lines were cultured and maintained in RPMI-1640 medium. And LLC-1 cells were cultured and maintained in DMEM

medium. Both media were supplemented with 10% fetal bovine serum (FBS) and 1% penicillin-streptomycin. All cell lines were cultured in a 37 °C humidified environment with 5% CO_2 supply.

BALB/c mice (6-8 weeks, female) were purchased from SiPeiFu Biotechnology Company (Beijing, China). All the animal experiments were approved by the Medical Ethics Committee of Sichuan University, and the animal experiments were performed in the Animal Laboratory of West China School of Pharmacy in Sichuan University (accreditation number: SYXK (Chuan)2018–113).

Preparation and characterization of zCT26-DBCO

CT26 cells (1 x 10^6) were treated with 50 μM oncolytic peptide LTX-315 (amino acid sequence: CKKWWKKW(Dip)K-NH_2, Shanghai Apeptide Co., Ltd. Shanghai, China) at 37 °C for 2 h. Then, the suspension was centrifuged, and cell pellet was washed with PBS thrice. Cell corpses succumbing to oncolysis were collected, and further inserted with dibenzocyclooctylenyl (DBCO) on cell surface after incubating with 100 μg/mL of 1,2-distearoyl-sn-glycero-3-phosphoethanolamine-N-amino(polyethylene glycol)2000-dibenzocyclooctylenyl (DSPE-PEG_{2k}-DBCO, Pansure Biotechnology, Shanghai, China) at 37 °C for 20 min. Afterward, the cell suspension was centrifuged, and cell pellet was washed with PBS thrice to collect zCT26-DBCO as final product.

To characterize the immunomodulatory signal emitting zCT26-DBCO, we measured the continuous cell-surface exposure of calreticulin (CRT) and adenosine triphosphate (ATP) at 0 h, 4 h, 8 h, and 24 h after suspending freshly made zCT26-DBCO in serum-free culture medium. For CRT measurement, after per-blocked with BSA (5%), zCT26-DBCO (1 x 10^6) was incubated with mouse antibody CRT (1:400 dilution, Abcam, Cambridge, UK) at 4 °C for 1 h, washed with PBS thrice, and further incubated with Alexa Fluor 647-conjugated secondary antibody (1:400 dilution, Abcam, Cambridge, UK) at 4 °C for 1 h, and then washed with PBS thrice, followed by flow cytometry (Beckman Coulter, California) analysis. For quantitative detection of ATP secretion, the supernatant of zCT26-DBCO suspension was collected for ATP concentration measurement by an ATP Assay Kit (Beyotime, Shanghai).

For the detection of DSPE-PEG$_{2k}$-DBCO inserted into cell corpses succumbing to oncolysis, DBCO-PEG$_{2k}$-Cy5 was substituted to represent DBCO-PEG$_{2k}$-DBCO. CT26 cancer cells (1 x 10^6) were incubated with LTX-315 (50 μM) at 37 °C for 2 h, washed with PBS thrice. Next, cells were incubated with DSPE-PEG$_{2k}$-Cy5 (100 μg/mL) at 37 °C for 20 min and then washed with PBS thrice to obtain zCT26 cells surface anchoring Cy5. Furthermore, after per-blocked with BSA (5%), zCT26 cells with surface anchoring Cy5 were incubated with the mouse antibody CRT (1:400 dilution) at 4 °C for 1 h, washed with PBS thrice and then stained with Alexa Fluor 555-conjugated mouse antibody (1:400 dilution, Abcam, Cambridge, UK) at 4 °C for 1 h, washed with PBS thrice. After that, cells were stained with 10 ng/mL of 4',6-Diamidino-2-phenylindole dihydrochloride (DAPI, Solarbio, Beijing, China) at room temperature for 10 min, washed with PBS thrice. Cell products were detected by confocal laser scanning microscopy (CLSM, Zeiss LSM510 DUE, Jena, Germany)

Preparation and characterization of azido-tagged 4T1 cells

4T1 cells or 4T1-EGFP were seeded in 35 mm glass culture dishes at a density of 1 x 10^5 cells and incubated with 50 μM of 1,3,4-*O*-acetyl-*N*-azidoacetylmannosamine (Ac$_4$ManNAz Aladdin Biochemical Technology Company, Shanghai, China) for 72 h to express azide groups on cell surface. Then the cells were washed with PBS thrice, and incubated with 50 μM of DBCO-terminated fluorophore (DBCO-Cy5, Aladdin Biochemical Technology Company, Shanghai, China) at 37 °C for 30 min to detect the azido expression. After that, cells were washed with PBS thrice and further stained with DAPI (10 ng/mL) at room temperature for 10 min, washed with PBS thrice, prior to confocal visualization.

Investigation of cell-cell contact in vitro

To investigate the bioorthogonal reaction between zCT26-DBCO and azido-tagged 4T1-EGFP (4T1-EGFP-N$_3$), an equal number of zCT26-DBCO pre-stained with DAPI (10 ng/mL, room temperature, 10 min) were mixed with 4T1-EGFP-N$_3$ at 37 °C for 1 h, followed by CLSM analysis. To further confirm the interaction of zCT26-DBCO and azido-tagged 4T1 (4T1-N$_3$) cells, 4T1-N$_3$ (1 x 10^6) and pre-made zCT26-DBCO (1 x 10^6) were mixed in cell culture medium at 37 °C for 1 h. Then, the cell clusters were sequentially fixed with 4%

paraformadldehyde at room temperature for 10 min, dehydrated in a series of graded ethanol (30%, 50%, 70%, 90% and 100%), dried by a critical point dryer, and surface coated with gold, prior to observation with scanning electron microscopy (SEM).

Investigation of pseudo-oncolysis in 4T1-N_3 anchored by zCT26-DBCO

CT26 cell corpses undergoing oncolysis (zCT26) and zCT26-DBCO (1 x 10^6) with CRT labeling as described above were pre-stained, tracked by fluorescent 1,1′-Dioctadecyl-3,3,3′,3′-tetramethylindodicarbocyanine, 4-chlorobenzenesulfonate salt (DiD, Solarbio, Beijing, China), and further incubated with equal number of 4T1-EGFP and 4T1-EGFP-N_3, respectively, at 37 °C for 1 h. Afterward, flow cytometry analysis was employed to quantify the cell-cell binding, and measure the intensity of CRT expressed by CT26 cell corpses in $EGFP^+$ gated cells.

For analysis of ATP sustained secretion, 4T1 cells were seeded into a 6 well plate at a density of 1 x 10^5 cells and incubated with $Ac_4ManNAz$ (50 μM) for 72 h to express azide groups on cell surface. Next, zCT26-DBCO (1 x 10^6) were pre-made and resuspended in fresh culture medium, added into the 6 well plate and incubated with adherent azido-tagged 4T1 cells at 37 °C for 1 h. Then, unbound zCT26-DBCO was washed off with PBS, and fresh culture medium was added to 6-well plates, and the secretion of ATP in the supernatant was measured by an ATP Assay Kit at 0 h, 4 h, 8 h, and 24 h.

Investigation of phagocytosis of 4T1-EGFP-N_3 anchored by zCT26-DBCO

To investigate the phagocytosis, bone marrow-derived dendritic cells (BMDCs) were isolated and cultured according to the protocol as previously reported.[1] Then, BMDCs were stained with 2 μg/mL of 1,1'-Dioctadecyl-3,3,3',3'-tetramethylindodicarbocyanine 4-chlorobenzenesulfonate (DiD, Solarbio, Beijing, China) at 37 °C for 30 min, washed with PBS thrice, and obtained as BMDC-DiD. After zCT26-DBCO (1 x 10^5) and 4T1-EGFP-N_3 (1 x 10^5) were mixed at 37 °C for 1 h, BMDC-DiD (1 x 10^5) were added to the mixture and further incubated at 37 °C for 4 h. For control groups, BMDC-DiD were directly incubated with 4T1-EGFP or cell mixture of 4T1-EGFP and CT26 corpses undergoing oncolysis (zCT26) with DBCO modification at 37 °C for 4 h. Then, the phagocytosis by BMDC were

quantified by flow cytometry analysis.

Preparation of local hydrogel delivery platform

The hydrogel crosslinker, ROS-liable *N*^{1}-(4-boronobenzyl)-*N*^{3} (4-boronophenyl)-*N*^{1}, N^1, *N*^{3}, *N*^{3}-tetramethylpropane-1,3-diaminium (TSPBA), was synthesized as previously reported.[2, 3] Briefly, *N*, *N*, *N′*, *N′*-tetramethyl-1,3-propanediamine (0.2 g, 1.5 mmol) and 4-(bromomethyl)phenylboronic acid (1 g, 4.6 mmol) were dissolved in dimethylformamide (DMF, 20 mL). After stirring at 60 °C for 12 h, the solution was added into tetrahydrofuran (THF) (100 mL) to precipitate, filtered, and washed with THF thrice (3 x 20 mL). The precipitation was collected by centrifugation (5000 rpm, 10 min, KDC-140HR, Anhui USTC Zonkia Scientific Instruments Co., Ltd., China). After drying under vacuum, 0.63 g purified TSPBA was obtained with a yield of 73.9% and characterized by ^{1}H NMR. ^{1}H nuclear magnetic resonance (300 MHz, d-DMSO, δ): 8.132 (s, 4H), 7.85 (d, 4H), 7.49 (d, 4H), 4.58 (s, 4H), 3.26 (s, 4H), 2.97 (s, 12H), 2.38 (m, 2H). The hydrogel precursor solution was prepared by dissolving 5 g poly(vinyl alcohol) (PVA, Aladdin Biochemical Technology Company, Shanghai, China) in 100 mL deionized water at 100 °C to acquire a PVA solution (5 wt%, ~ 75 kDa, 99% hydrolyzed).

Blank Gel was immediately formed when mixing 100 μL of PVA solution with an equal volume of TSPBA solution (3 mg in 100 μL of H_2O). For preparation of D-Gel, 0.2 mg of dexamethasone (Yuanye Biotechnology, Shanghai, China) was dissolved in 40 μL of PEG-400 solution and then mixed with 60 μL of TSPBA solution to form a crosslinker solution. Then, 100 μL of PVA solution was added to an equal volume of the crosslinker solution to obtain D-Gel.

$AlCV_{CT26}$@D-Gel/ $AlCV_{LLC-1}$@D-Gel/ $AlCV_{B16}$@D-Gel involved two-step gelation. The first step hydrogel was D-Gel prepared as described above. The second step hydrogel was allogeneic cell-laden hydrogel, in which CT26/LLC-1/B16 cell corpses (1 x 10^6) succumbing to oncolysis were dispersed into 100 μL of PVA solution, and then added into equal volume of TSPBA solution to transition from solution to hydrogel.

Similarly, $AuCV_{4T1}$@D-Gel involved two-step gelation. The first step hydrogel was D-Gel

prepared as described above. The second step hydrogel was autologous cell-laden hydrogel, in which 4T1 cell corpses (1 x 10^6) succumbing to oncolysis were dispersed into 100 μL of PVA solution, and then added into equal volume of TSPBA solution to transition from solution to hydrogel.

Zombie cell vaccine platforms of ZCV_{CT26}@D-Gel, ZCV_{LLC-1}@D-Gel, and ZCV_{B16}@D-Gel involved two-step gelation as well. For the preparation of the first step hydrogel, 0.2 mg dexamethasone was dissolved in 40 μL of PEG-400 solution, and then mixed with 60 μL of H_2O dissolving 3 mg of TSPBA and 0.4 mg of $Ac_4ManNAz$ to form a crosslinker solution. Dexamethasone and $Ac_4ManNAz$ co-loaded hydrogel was immediately formed upon mixture of crosslinker solution and PVA solutions. For the preparation of the second step hydrogel, DBCO-modified LLC-1/B16 cell corpses succumbing to oncolysis were fabricated following an identical protocol of preparing zCT26-DBCO as described above. Then premade zCT26-DBCO, zB1-DBCO, or zLLC-1-DBCO (1 x 10^6) were dispersed into 100 μL of PVA solution, and then mixed with equal volume of TSPBA solution to transition from solution to hydrogel.

Analogous cell vaccine platform of poor immunogenicity was prepared as LNT-CV_{CT26}@D-Gel, which involved two-step gelation as well. The first step hydrogel was dexamethasone and $Ac_4ManNAz$ co-loaded hydrogel prepared as described above. For the preparation of the second step hydrogel, CT26 cells (1 x 10^6) were suspended into the cell cryopreservation medium, immersed in liquid nitrogen for 12 h, and then thawed at 37 °C. After washed with PBS, the cell corpses were incubated with DSPE-PEG_{2k}-DBCO (100 μg/mL) at 37 °C for 20 min to fabricate DBCO-modified and liquid nitrogen–treated CT26 cell corpses. Then, the premade cell products were dispersed into 100 μL of PVA solution, and then mixed with equal volume of TSPBA solution to transition from solution to hydrogel.

Characterization of local hydrogel delivery system

The morphology of the freeze-dried blank hydrogel and hydrogel precursor were observed by scanning electron microscope (SEM, SU3500/Aztec X-Max20). Zombie cells-loaded hydrogel was characterized by CLSM. Specifically, CT26 cells (1 x 10^6) were co-cultured with LTX-315 (50 μM) at 37 °C for 2 h, and then were incubated with fluorescent

DSPE-PEG_{2k}-Cy5 (100 μg/ml) at 37 °C for 20 min to produce zCT26 cells with surface anchoring Cy5. Next, after per-blocked with BSA (5%), cells were incubated with the mouse antibody CRT (1:400 dilution) at 4 °C for 1 h, washed with PBS thrice and then stained with Alexa Fluor 647-conjugated antibody (1:400 dilution) at 4 °C for 1 h, and washed with PBS thrice. Next, cells were stained with DAPI (2 μg/mL) at room temperature for 10 min and then washed with PBS thrice. Furthermore, 1 x 10^6 zCT26 stained with CRT, Cy5 and DAPI were loaded into hydrogel, and a three-dimension construction image of hydrogel were detected by CLSM.

To verify rheological behavior, frequency-dependent rheological properties of PVA precursor, blank hydrogel, small molecular payloads (i.e., 0.2 mg dexamethasone and 0.4 mg Ac_4ManNAz) loaded hydrogel and zCT26-DBCO (1 x 10^6) loaded hydrogel were measured at room temperature with a shear strain of 1.00% and an oscillation frequency of 0.1 to 10 rad/s by TA Instruments AR 2000 rheometer (MCR302, Anton Paar).

To investigate the ROS-responsive degradation and payload release behaviors of hydrogels in vivo, 4T1 cells (1 × 10^6) in 100 μL of PBS were implanted in the breast pad of BALB/c mice (6-8 weeks, female) on day -12. Next, we performed tumor removal surgery on BALB/c mice with orthotopic 4T1 tumors, intentionally leaving ~5% of tumor behind on day 0, and then dexamethasone (0.2 mg) and Ac_4ManNAz (0.4 mg) loaded hydrogels were immediately injected into surgical bed, and the size of dexamethasone plus Ac_4ManNAz co-loaded hydrogels were observed on day 0, day 0.5, day 2 and day 4 after tumor resection. And healthy mice were subcutaneously injected with D-Gel as control group. Besides, pre-made zCT26-DBCO (1 x 10^6) loaded hydrogels were injected into surgical bed on day 4 after tumor resection when D-Gel degraded completely and ZCV-Gel size was recorded on day 0, day 0.5, day 2 and day 4 after ZCV-Gel was injected. The size of hydrogels was photographed on the last day for recording.

Furthermore, to verify the sustained release of encapsulated cargos in hydrogel, fluorescent cyanine 5 (Cy5, 2 nM) was loaded into hydrogel and immediately injected into surgical cavity after tumor resection. As control groups, Cy5 (2 nM) loaded hydrogels and free Cy5 (2 nM) were injected into normal subcutaneous tissues and surgical bed,

respectively. Furthermore, the fluorescence images of Cy5 were recorded through an IVIS Spectrum In Vivo Imaging System (PerkinElmer, Lumina 3) on day 0, day 0.5, day 2, and day 4 after anaesthetized by ethobrom.

Investigation of cell-cell binding in vivo

To investigate the interaction of zCT26-DBCO and azido-tagged 4T1 residual tumor cells in vivo, zCT26-DBCO (1 x 10^6) stained with carboxymethyl fluorescein diacetate (CMFDA, 2 μg/mL) was loaded into hydrogel. 4T1 cells (1 × 10^6) in 100 μL of PBS were implanted in the breast pad of BALB/c mice (6-8 weeks, female) on day -12. Next, we performed tumor removal surgery on BALB/c mice with orthotopic 4T1 tumors intentionally leaving ~5% of tumor behind on day 0, and $Ac_4ManNAz$ (0.4 mg) loaded hydrogels were immediately injected into surgical bed to modify residual tumor cells with azide groups. On day 4, CMFDA-tracked zCT26-DBCO (1 x 10^6) loaded hydrogels were injected into surgical bed. Mouse were sacrificed and tumor tissues were collected on day 6 after tumor resection. For the CLSM analysis, frozen tumor sections (10 μm) were made and stained with DAPI (10 ng/mL) at room temperature for 10 min, washed with PBS thrice and further incubated with DBCO-Cy5 (50 μM) to stain cell surface expressed azide groups at 37 °C for 30 min, washed with PBS thrice, prior to confocal visualization.

Furthermore, collected tumor tissues were cut into small pieces, and tumor cells were dissociated in digestion buffer. And the cell suspension was passed through a 70 μm nylon strainer to get the single cell suspensions, and then washed with PBS thrice, incubated with ACK lysing buffer (4 °C, 10 min, Solarbio) to lyse red blood cells. After washed thrice with cold PBS, tumor cells were resuspended in PBS buffer. Next, single cell suspensions were incubated with DBCO-Cy5 (50 μM) to stain cell surface expressed azide groups at 37 °C for 30 min. Afterward, flow cytometry analysis was used to compare the binidng of CMFDA-labeled zCT26-DBCO to azido-positive and negative cell subpopulations.

Evaluation of safety profile of delivery system

BALB/c mice (6-8 weeks, female) were randomly divided into six groups as follow: (1) Saline, and (2) ZCV_{CT26}@D-Gel. The mice were subcutaneously inoculated with 1 x 10^6

4T1 cells at the ventral mammary fat pad on day -12. When the tumor volume increased to about 200 mm^3 on day 0, tumors were resected and maintained about 5% residual tumor tissue and received treatment as described above. On day 18 after tumor resection, blood, host skin tissues surrounding the hydrogel and major organs (heart, spleen, liver and kidney) were collected from each mouse.

The supernatant (serum) of blood was sent to State/National Key Laboratory of Biotherapy for serum chemistry analysis. The whole blood in dipotassium EDTA tube were sent to West China School of Pharmacy for leukocytes, erythrocytes, and thrombocytes analysis.

The host skins and major major organs (heart, spleen, liver and kidney) were sent to Lilai biomedicine experiment center for hematoxylin and eosin (H&E) staining.

Evaluation of anti-tumor efficacy in vivo

BALB/c mice (6-8 weeks, female) were randomly divided into six groups as follow: (1) Saline, (2) Blank Gel, (3) D-Gel, (4) $AlCV_{CT26}$@D-Gel, (5) ZCV_{CT26}@D-Gel, (6) ZCV_{CT26}@D-Gel + CD8 depletion. The mice were subcutaneously inoculated with 1 x 10^6 4T1 cells suspended in 100 μL of PBS at the ventral mammary fat pad on day -12. When the tumor volume increased to about 200 mm^3 on day 0, tumors were resected and maintained about 5% residual tumor tissue and received treatment. Specifically, ZCV_{CT26}@D-Gel was a stepwise hydrogel, we firstly injected dexamethasone (0.2 mg) and $Ac_4ManNAz$ (0.4 mg) loaded hydrogels into surgical bed on day 0 after tumor resection, and pre-made zCT26-DBCO (1 x 10^6) were loaded into hydrogels and injected into surgical cavity on day 4. For ZCV_{CT26}@D-Gel + CD8 depletion group, mice received ZCV_{CT26}@D-Gel treatment was intraperitoneally injected with the mouse CD8 monoclonal antibody (100 μg, Leinco Technologies, Inc.) on day 5. For $AlCV_{CT26}$@D-Gel group, D-Gel (0.2 mg dexamethasone equivalence) was injected into surgical bed on day 0, and then hydrogel loaded with pre-made 1 x 10^6 CT26 cell corpses succumbing to the oncolysis loaded was injected into surgical cavity on day 4. Besides, for D-Gel (0.2 mg dexamethasone equivalence) and Blank Gel groups, hydrogels were injected into surgical bed on day 0 immediately after tumor resection. Tumor volumes, body weights and survival rates were recorded every

other day by vernier calipers, and tumor volume (mm^3) was calculated as (long diameter × short diameter^2)/2. Mice were euthanized when they lost more than 20% of their body weight or when their tumor volume exceeded 1500 mm^3.

Immune Status Investigation

BALB/c mice (6-8 weeks, female) were randomly divided into four groups as follow: (1) Untreated, (2) D-Gel, (3) $AlCV_{CT26}$@D-Gel, (4) ZCV_{CT26}@D-Gel. The orthotopic breast tumor recurrence models after incomplete tumor resection was established and various hydrogel formulations were locally injected as described above. Then, the recurrent tumors were collected from each mouse on day 14 after tumor resection. Collected tumor tissues were cut into small pieces, and tumor cells were dissociated in digestion buffer. And the cell suspension was passed through a 70 μm nylon strainer to obtain the single cell suspensions, and then washed with PBS thrice, incubated with ACK lysing buffer (4 °C, 10 min) to lyse red blood cells. After washed thrice with cold PBS, tumor cells were resuspended in PBS buffer. Furthermore, the single-cell suspensions were fixed with 4% paraformaldehyde for 15 min, and washed with PBS thrice. After per-blocked with BSA (5%), the single-cell suspensions were stained with FITC-conjugated mouse antibody CD3 (1:200 dilution, Biolegend, San Diego, CA, USA), and APC-conjugated mouse antibody CD8 (1:200 dilution, Biolegend, San Diego, CA, USA) at 4 °C for 1 h, and then washed with PBS thrice. After that, these cells were punched and the stained with PE-conjugated mouse antibody IFN-γ (1:200 dilution, Biolegend, San Diego, CA, USA) at 4 °C for 1 h, and then washed with PBS thrice, prior to flow cytometry analysis. To detect the COX-2 expression in tumor, cells were punched and then stained with mouse antibody COX-2 (1:200 dilution, Affinity Biosciences, Jiangsu, China) at 4 °C for 1 h, and washed with PBS thrice, followed by co-culturing with Alexa Fluor 647-conjugated secondary antibody (1:200 dilution) at 4 °C for 1 h, and further washed with PBS thrice for flow cytometry analysis. To detect the intratumoral level of PGE2, cell suspensions before ACK lysing were detected by a mouse PGE2 ELISA kit (Shanghai Enzyme-linked Biotechnology) after centrifugation and collecting the supernatant. To identify MDSCs ($CD11b^+Gr1^+$), cell suspensions were stained with FITC-conjugated mouse antibody Gr1 (1:200 dilution) and PE-conjugated mouse antibody

CD11b (1:200 dilution, eBioscience, Carlsbad, CA, USA) at 4 °C for 1 h. To identify TAMs ($CD11b^+F4/80^+CD206^{hi}$), cell suspensions were stained with PE-conjugated mouse antibody CD11b (1:200 dilution), FITC-conjugated mouse antibody F4/80 (1:200 dilution), and APC-conjugated mouse antibody CD206 (1:200 dilution).

Evaluation of mature dendritic cells in tumor-draining lymph nodes

BALB/c mice (6-8 weeks, female) were randomly divided into four groups as follow: (1) Untreated, (2) D-Gel, (3) LNT-CV_{CT26}@D-Gel, (4) ZCV_{CT26}@D-Gel. 21 days after tumor resection and the treatments, tumor-draining lymph nodes were collected, and cut into small pieces, and cells were dissociated in digestion buffer. And the cell suspensions were passed through a 70 μm nylon strainer to get the single cell suspensions, and then washed with PBS thrice. Then, after per-blocked with BSA (5%), the single-cell suspensions were stained with FITC-conjugated mouse antibody CD11c (1:200 dilution, eBioscience, Carlsbad, CA, USA), PE-conjugated mouse antibody CD80 (1:200 dilution, eBioscience, Carlsbad, CA, USA) and APC-conjugated mouse antibody CD86 (1:200 dilution, eBioscience, Carlsbad, CA, USA) at 4 °C for 1 h. Afterward, cells were washed with PBS thrice prior to flow cytometry analysis.

Evaluation of activation of immune effector memory T cells in spleen

21 days after tumor resection and the treatments, the spleens were collected, and cut into small pieces, and tumor cells were dissociated in digestion buffer. And the cell suspensions were passed through a 70 μm nylon strainer to get the single cell suspensions, and then washed with PBS thrice, incubated with ACK lysing buffer (4 °C, 10 min) to lyse red blood cells. After washed thrice with cold PBS, cells were resuspended in PBS buffer. After that, single-cell suspensions were incubated with BSA (5%) and then stained with PerCP-Cy5.5-conjugated mouse antibody CD62L (1:200 dilution, Biolegend, San Diego, CA, USA), PE-conjugated mouse antibody CD44 (1:200 dilution, Biolegend, San Diego, CA, USA) and APC-conjugated mouse antibody CD8 (1:200 dilution) at 4 °C for 1 h to identify effector memory T cells in spleen by flow cytometry analysis.

Evaluation of generation of systemic immune memory

BALB/c mice (6-8 weeks, female) were randomly divided into three groups as follow: (1) Untreated, (2) $AlCV_{CT26}$@D-Gel, (3) ZCV_{CT26}@D-Gel. The mice were subcutaneously inoculated with 1 x 10^6 4T1 cells at the ventral mammary fat pad on day -12. When the tumor volume increased to about 200 mm^3 on day 0, tumors were resected and maintained about 5% residual tumor tissue and received treatment as described above. On day 4, 4T1-Luc cells (1 × 10^6) were intravenously injected into treated mice. After that, bioluminescence images were used to monitor the metastasis of 4T1-Luc cells on day 7, day 11 and day 15 by using an IVIS Spectrum In Vivo Imaging System 10 min after mice were intraperitoneally injected with D-luciferin potassium salt (15 mg/mL, 200 μL, Energy Chemical, Shanghai, China).

On day 16, mice were sacrificed, peripheral blood was collected, and peripheral blood mononuclear cells (PBMCs) were isolated using Ficoll Plus 1.084 (Solarbio, Beijing, China). PBMCs were further purified by incubating with ACK lysing buffer (4 °C, 10 min) to lyse red blood cells. Next, PBMCs (1 x 10^6) were prepared in 10% FBS fresh cell culture medium and then co-cultured with 0.1 million live 4T1 cells per well at 37 °C overnight in the presence of the protein transport inhibitor, monensin (1 μM). Next, after per-blocked with BSA (5%), PBMCs were incubated with APC-conjugated mouse antibody CD8 (1:200 dilution), and washed with PBS thrice. Then, these cells were punched and the stained with PE-conjugated mouse antibody IFN-γ (1:200 dilution) at 4 °C for 1 h, and then washed with PBS thrice, followed by flow cytometry analysis.

Evaluation of generation of adaptive immunity against 4T1 tumors

BALB/c mice (6-8 weeks, female) were randomly divided into six groups as follow: (1) Untreated, (2) $AlCV_{CT26}$@D-Gel, (3) ZCV_{CT26}@D-Gel. The mice were subcutaneously inoculated with 1 x 10^6 4T1 cells at the ventral mammary fat pad on day -12. When the tumor volume increased to about 200 mm^3 on day 0, tumors were resected and maintained about 5% residual tumor tissue and received treatment as described above. On day 7 and day 15 after tumor resection, mice were sacrificed and tumor were collected. Collected tumor tissues were cut into small pieces, and tumor cells were dissociated in digestion buffer. And the cell suspensions were passed through a 70 μm nylon strainer to get the

single cell suspensions, and then washed with PBS thrice, incubated with ACK lysing buffer (4 °C, 10 min) to lyse red blood cells. After washed thrice with cold PBS, cells were resuspended in PBS buffer.

To detect the tumor-infiltrated T cells, after per-blocked with BSA (5%), the single-cell suspensions were stained with FITC-conjugated mouse antibody CD3 (1:200 dilution), PE/Cyanine7-conjugated mouse antibody CD4 (1:200 dilution), and APC-conjugated mouse antibody CD8 (1:200 dilution) at 4 °C for 1 h. Then, these cells were punched and the stained with PE-conjugated mouse antibody IFN-γ (1:200 dilution) at 4 °C for 1 h, and then washed with PBS thrice, followed by flow cytometry analysis.

To detect the tumor-infiltrated plasmacytoid dendritic cells ($CD11c^+SiglecH^+$) and myeloid dendritic cells ($CD11c^+CD80^+$, $CD11c^+MHCII^+$), after per-blocked with BSA (5%), the single-cell suspensions were stained with APC-conjugated mouse antibody CD11c (1:200 dilution), PE-conjugated mouse antibody SiglecH (1:200 dilution), PerCP-Cy5.5-conjugated mouse antibody MHCII (1:200 dilution) and FITC-conjugated mouse antibody CD80 (1:200 dilution) at 4 °C for 1 h, and then washed with PBS thrice and all samples were detected by flow cytometry analysis**.**

To detect the tumor-infiltrated natural killer cells ($CD3^-CD49b^+$), after per-blocked with BSA (5%), the single-cell suspensions were stained with FITC-conjugated mouse antibody CD3 (1:200 dilution), and PE -conjugated mouse antibody CD49b (1:200 dilution) at 4 °C for 1 h, and then washed with PBS thrice and all samples were detected by flow cytometry analysis**.**

Evaluation of general applicability of the "zombie" technique from other allogeneic cell sources

BALB/c mice (6-8 weeks, female) were randomly divided into six groups as follow: (1) $AuCV_{4T1}$@D-Gel, (2) ZCV_{B16}@D-Gel, (3) $AlCV_{B16}$@D-Gel, (4) ZCV_{LLC-1}@D-Gel, (5) $AlCV_{LLC-1}$@D-Gel, (6) left untreated. The mice were subcutaneously inoculated with 1 x 10^6 4T1 cells at the ventral mammary fat pad on day -12. When the tumor volume increased to about 200 mm^3 on day 0, tumors were resected and maintained about 5% residual tumor

tissue and received treatment immediately. Specifically, following an identical procedure ZCV_{CT26}@D-Gel, ZCV_{B16}@D-Gel/ ZCV_{LLC-1}@D-Gel involved administering a dexamethasone (0.2 mg) plus $Ac_4ManNAz$ (0.4 mg) co-loaded hydrogel on day 0, followed by a hydrogel loaded with DBCO-modified B16/LLC-1 cell corpses (1 x 10^6) succumbing to oncolysis on day 4. $AlCV_{B16}$@D-Gel/ $AlCV_{LLC-1}$@D-Gel/ $AuCV_{4T1}$@D-Gel involved administrating a dexamethasone (0.2 mg) loaded hydrogel on day 0, followed by a hydrogel loaded with B16/LLC-1/4T1 cell corpses (1 x 10^6) succumbing to oncolysis on day 4. Similarly, $AuCV_{4T1}$@D-Gel was involved administering a dexamethasone (0.2 mg) loaded hydrogel on day 0, followed by a hydrogel loaded with 4T1 cell corpses (1 x 10^6) succumbing to oncolysis on day 4. Moreover, tumor volume was recorded every two days until mice were sacrificed on day 22, and the tumors were collected from each mouse. Collected tumor tissues were cut into small pieces, and tumor cells were dissociated in digestion buffer. And the cell suspensions were passed through a 70 μm nylon strainer to get the single cell suspensions, and then washed with PBS thrice, incubated with ACK lysing buffer (4 °C, 10 min) to lyse red blood cells. After washed thrice with cold PBS, cells were resuspended in PBS buffer. After per-blocked with BSA (5%), the single-cell suspensions were stained with FITC-conjugated mouse antibody CD3 (1:200 dilution), and APC-conjugated mouse antibody CD8 (1:200 dilution) at 4 °C for 1 h, and then washed with PBS thrice. After that, these cells were punched and the stained with PE-conjugated mouse antibody IFN-γ (1:200 dilution) at 4 °C for 1 h. All samples were detected by flow cytometry analysis. At endpoint, PBMCs were prepared as described above, and then co-cultured with 0.1 million live 4T1 cells per well at 37 °C overnight in the presence of the protein transport inhibitor, monensin (1 μM). Next, after per-blocked with BSA (5%), PBMCs were incubated with APC-conjugated mouse antibody CD8 (1:200 dilution), and washed with PBS thrice. Then, these cells were punched and the stained with PE-conjugated mouse antibody IFN-γ (1:200 dilution) at 4 °C for 1 h, and then washed with PBS thrice, followed by flow cytometry analysis.

Evaluation of long-term anti-tumor efficacy in the post-operative model of subcutaneous CT26 colon cancer

BALB/c mice (6-8 weeks, female) were randomly divided into six groups as follow: (1) left untreated, (2) $AlCV_{4T1}$@D-Gel (×1, single dose), (3) ZCV_{4T1}@D-Gel (×1, single dose), (4) LTX-315+D-Gel, (5) ZCV_{4T1}@D-Gel (×3, three-cycle treatment biweekly), and (6) $AuCV_{CT26}$@D-Gel (×3, three-cycle treatment biweekly). The mice were subcutaneously inoculated with 1 x 10^6 CT26 cells suspended in 100 μL of PBS were subcutaneously injected into mice on the right flank. When the tumor volume increased to about 200 mm^3 on day 0, tumors were resected and maintained about 5% residual tumor tissue and received treatment. Among these groups, $AlCV_{4T1}$@D-Gel represents an allogeneic cell vaccine strategy which involved administrating a dexamethasone loaded hydrogel in resection cavity immediately after surgery, followed by a hydrogel encapsulating 4T1 cell corpses, pretreated with LTX-315 and undergoing oncolysis, four days later; ZCV_{4T1}@D-Gel represents a zombie cell vaccine strategy in which dexamethasone and Ac_4ManNAz co-loaded hydrogel was given on day 0, and hydrogel encapsulating 4T1 cell corpses, undergoing oncolysis and modified with surface DBCO, was administered on day 4; $AuCV_{CT26}$@D-Gel represents an autologous cell vaccine strategy which involved administrating a dexamethasone loaded hydrogel on day 0, followed by a hydrogel loaded with CT26 cell corpses undergoing oncolysis on day 4; LTX-315+D-Gel represents an in situ vaccine strategy, where only visible only visible tumor remnants after surgery were treated by intratumoral injection of oncolytic peptide LTX-315, followed by immediate implantation of a dexamethasone loaded hydrogel.

Tumor volumes, and survival rates were recorded every other day by vernier calipers, and tumor volume (mm^3) was calculated as (long diameter × short diameter^2)/2. Mice were euthanized when they lost more than 20% of their body weight or when their tumor volume exceeded 1500 mm^3.

Evaluation of long-term anti-tumor efficacy in the post-operative model of orthotopic 4T1 breast cancer

BALB/c mice (6-8 weeks, female) were randomly divided into six groups as follow: (1) left untreated, (2) $AlCV_{CT26}$@D-Gel (×1, single dose), (3) ZCV $_{CT26}$@D-Gel (×1, single dose), (4) LTX-315+D-Gel, (5) ZCV $_{CT26}$@D-Gel (×3, three-cycle treatment biweekly), and (6)

$AuCV_{4T1}$@D-Gel (×3, three-cycle treatment biweekly). The mice were subcutaneously inoculated with 1 x 10^6 4T1 cells at the ventral mammary fat pad on day -12. When the tumor volume increased to about 200 mm^3 on day 0, tumors were resected and maintained about 5% residual tumor tissue and received treatment immediately.

Among these groups, $AlCV_{CT26}$@D-Gel represents an allogeneic cell vaccine strategy which involved administrating a dexamethasone loaded hydrogel in resection cavity immediately after surgery, followed by a hydrogel encapsulating 4T1 cell corpses, pretreated with LTX-315 and undergoing oncolysis, four days later; ZCV_{CT26}@D-Gel represents a zombie cell vaccine strategy in which dexamethasone and Ac_4ManNAz co-loaded hydrogel was given on day 0, and hydrogel encapsulating 4T1 cell corpses, undergoing oncolysis and modified with surface DBCO, was administered on day 4; $AuCV_{4T1}$@D-Gel represents an autologous cell vaccine strategy which involved administrating a dexamethasone loaded hydrogel on day 0, followed by a hydrogel loaded with CT26 cell corpses undergoing oncolysis on day 4; LTX-315+D-Gel represents an in situ vaccine strategy, where only visible only visible tumor remnants after surgery were treated by intratumoral injection of oncolytic peptide LTX-315, followed by immediate implantation of a dexamethasone loaded hydrogel.

Tumor volumes, and survival rates were recorded every other day by vernier calipers, and tumor volume (mm^3) was calculated as (long diameter × short diameter^2)/2. Mice were euthanized when they lost more than 20% of their body weight or when their tumor volume exceeded 1500 mm^3.

Evaluation of immune status in the post-operative model of subcutaneous CT26 colon cancer

BALB/c mice (6-8 weeks, female) were randomly divided into six groups as follow: (1) left untreated, (2) $AlCV_{4T1}$@D-Gel, and (3) ZCV_{4T1}@D-Gel. The mice were subcutaneously inoculated with 1 x 10^6 CT26 cells suspended in 100 μL of PBS were subcutaneously injected into mice on the right flank. When the tumor volume increased to about 200 mm^3 on day 0, tumors were resected and maintained about 5% residual tumor tissue and received treatment.

On day 24 after tumor resection, mice were sacrificed and tumor were collected. Collected tumor tissues were cut into small pieces, and tumor cells were dissociated in digestion buffer. And the cell suspensions were passed through a 70 μm nylon strainer to get the single cell suspensions, and then washed with PBS thrice, incubated with ACK lysing buffer (4 °C, 10 min) to lyse red blood cells. After washed thrice with cold PBS, cells were resuspended in PBS buffer.

To detect the tumor-infiltrated T cells, after per-blocked with BSA (5%), the single-cell suspensions were stained with FITC-conjugated mouse antibody CD3 (1:200 dilution), PE/Cyanine7-conjugated mouse antibody CD4 (1:200 dilution), and APC-conjugated mouse antibody CD8 (1:200 dilution) at 4 °C for 1 h. Then, these cells were punched and the stained with PE-conjugated mouse antibody IFN-γ (1:200 dilution) and Percp/5.5-conjugated mouse antibody Foxp3 at 4 °C for 1 h, and then washed with PBS thrice, followed by flow cytometry analysis.

Evaluation of immune status in the post-operative model of orthotopic 4T1 breast cancer

BALB/c mice (6-8 weeks, female) were randomly divided into six groups as follow: (1) left untreated, (2) AlCV$_{CT26}$@D-Gel, and (3) ZCV$_{CT26}$@D-Gel. The mice were subcutaneously inoculated with 1 x 10^6 4T1 cells suspended in 100 μL of PBS were subcutaneously injected into mice on the right flank. When the tumor volume increased to about 200 mm^3 on day 0, tumors were resected and maintained about 5% residual tumor tissue and received treatment.

On day 18 after tumor resection, mice were sacrificed and tumor were collected. Collected tumor tissues were cut into small pieces, and tumor cells were dissociated in digestion buffer. And the cell suspensions were passed through a 70 μm nylon strainer to get the single cell suspensions, and then washed with PBS thrice, incubated with ACK lysing buffer (4 °C, 10 min) to lyse red blood cells. After washed thrice with cold PBS, cells were resuspended in PBS buffer.

To detect the tumor-infiltrated T cells, after per-blocked with BSA (5%), the single-cell

suspensions were stained with FITC-conjugated mouse antibody CD3 (1:200 dilution), PE/Cyanine7-conjugated mouse antibody CD4 (1:200 dilution), and APC-conjugated mouse antibody CD8 (1:200 dilution) at 4 °C for 1 h. Then, these cells were punched and the stained with PE-conjugated mouse antibody IFN-γ (1:200 dilution) and Percp/5.5-conjugated mouse antibody Foxp3 (1:200 dilution) at 4 °C for 1 h, and then washed with PBS thrice, followed by flow cytometry analysis.

Supporting Figures and Tables

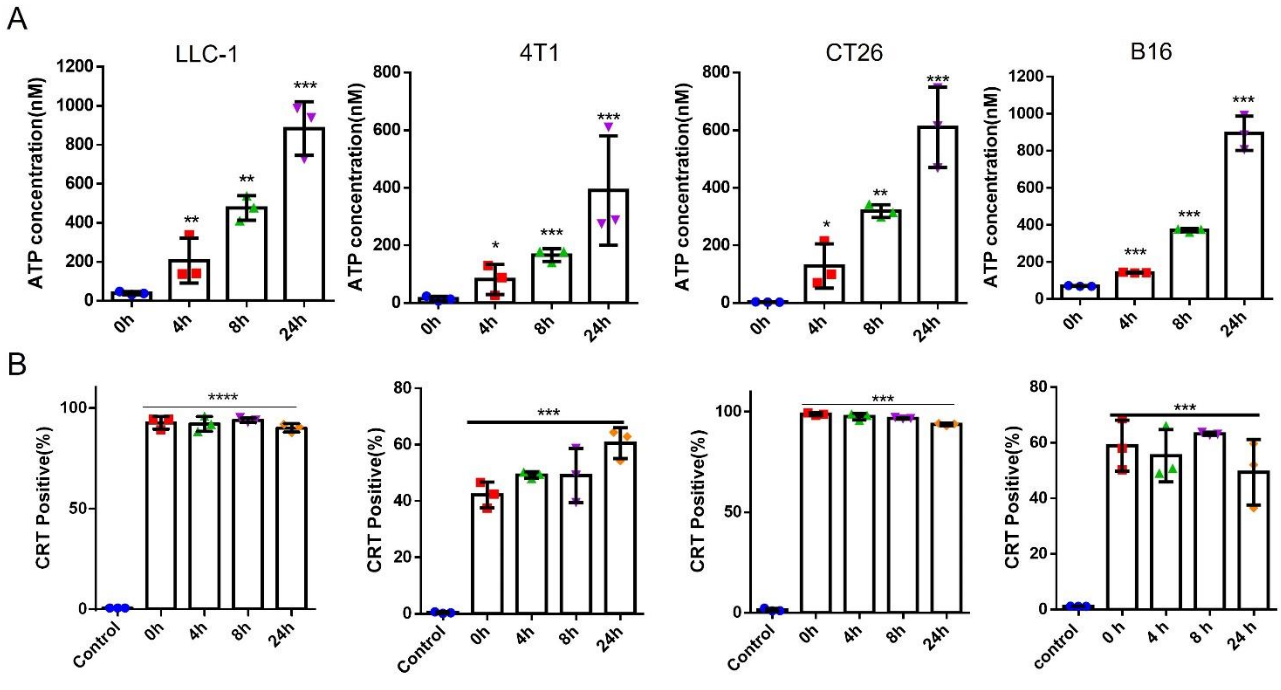

Figure S1. Characterization of cancer cell vaccines. LLC-1/4T1/CT26/B16 cells were first treated with a clinical-grade oncolytic peptide of LTX-315 at 37 °C for 2 h, and incubated with DSPE-PEG$_{2k}$-DBCO at 37 °C for 20 min to obtain zombie cell corpses. After zombie cell corpses were suspended in cell culture medium, continuous surface exposure of ATP (A) and extracellular release of adenosine CRT (B) over time were measured (n=3). Data are shown

as mean ± SD; *p < 0.05, **p <0.01, ***P < 0.001.

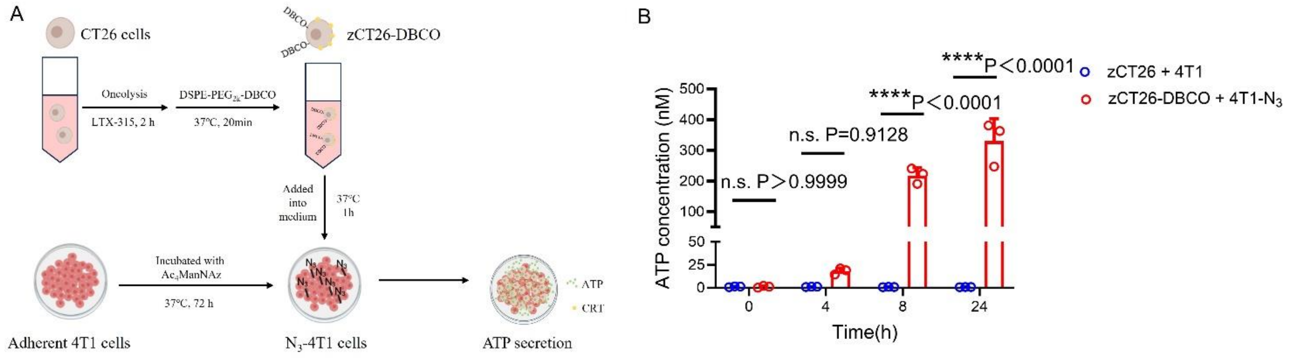

Figure S2. Zombie CT26 cells endowed azide groups modified 4T1 cells with ATP signals. (A) Illustration of zCT26-DBCO endowed targets with ATP signals. After incubated with LTX-315 (50 μM) at 37 °C for 2 h, CT26 cell corpses were further incubated with DSPE-PEG$_{2k}$-DBCO at 37 °C for 20 min to obtain zCT26-DBCO. Besides, 4T1 cells were seeded into glass culture dishes at a density of 1 x 10^5 cells and incubated with Ac_4ManNAz (50 μM) for 72 h to express azide groups on cell surface. Next, zCT26-DBCO resuspended into flesh culture medium was added into glass culture dishes to incubate with azido-tagged 4T1 cells at 37 °C for 1 h. Then, unbound zCT26-DBCO washed off by PBS. After 0, 4, 8, 24 h, ATP released in the supernatant was collected for detection. (B) ATP secretion was analyzed by ATP Assay Kit (n=3). Data are shown as mean ± SD; ****P < 0.0001.

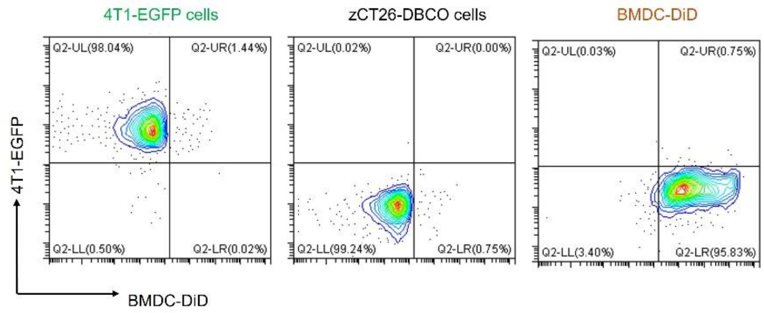

Figure S3. Single-component controls of 4T1-EGFP, zCT26 and BMDC-DiD.

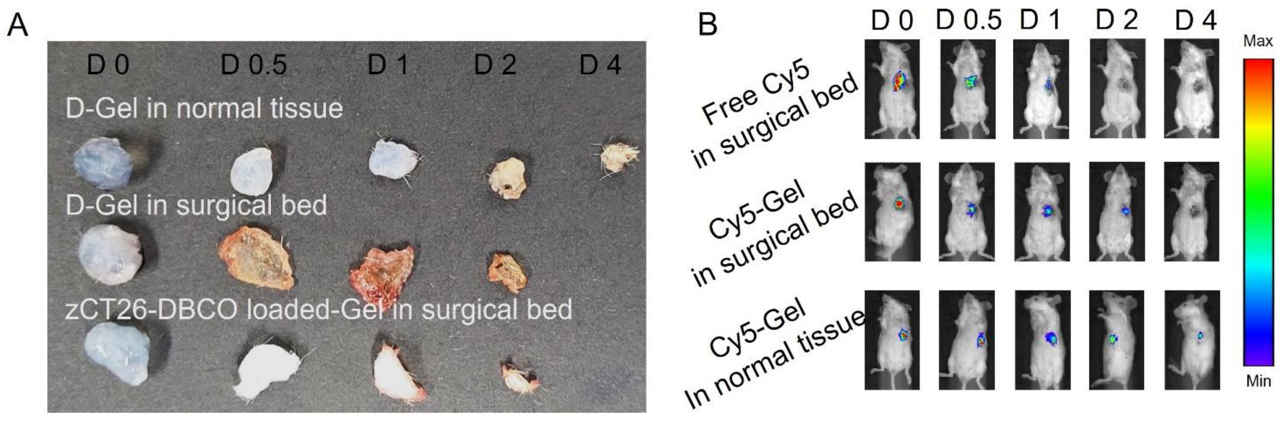

Figure S4. Hydrogel degradation in vivo. (A) Dexamethasone (0.2 mg) plus $Ac_4ManNAz$ (0.4 mg) co-loaded hydrogels (D-Gel) were immediately injected into surgical bed, and the size of dexamethasone plus $Ac_4ManNAz$ co-loaded hydrogels were observed on day 0, day 0.5, day 2 and day 4 after tumor resection. And healthy mice were subcutaneously injected with D-Gel as control group. Besides, zCT26-DBCO (1 x 10^6) loaded hydrogels were injected into surgical bed on day 4 after tumor resection when D-Gel degraded completely and zCT26-DBCO loaded hydrogels size was recorded on day 0, day 0.5, day 2 and day 4 after hydrogels were injected. The size of hydrogels was photographed on the last day for recording. (B) Fluorescent Cy5 was loaded into hydrogel and immediately injected into surgical cavity after tumor resection. As control groups, Cy5 loaded hydrogels and free Cy5 were injected into normal subcutaneous tissues and surgical bed, respectively. Furthermore, the fluorescence images of Cy5 were recorded through an IVIS Spectrum In Vivo Imaging System on day 0, day 0.5, day 2, and day 4.

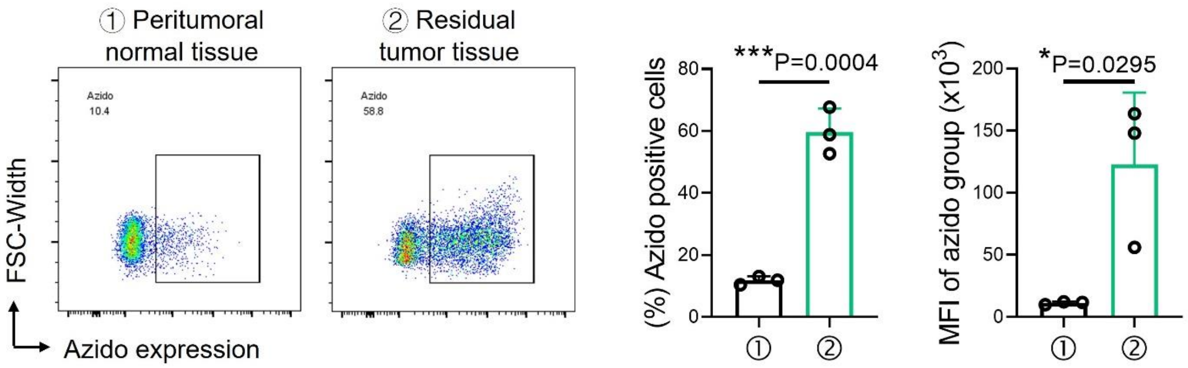

Figure S5. The evaluation of the off-target effect of $Ac_4ManNAz$-loaded hydrogel in post-surgical tumor bed. After tumor resection, $Ac_4ManNAz$-loaded hydrogels were

injected into resection cavity. Residual tumor tissues and peritumoral tissues were collected on day 4. Cells from these tissues were isolated and stained with DBCO-Cy5 to analyze the level of azido group expression.

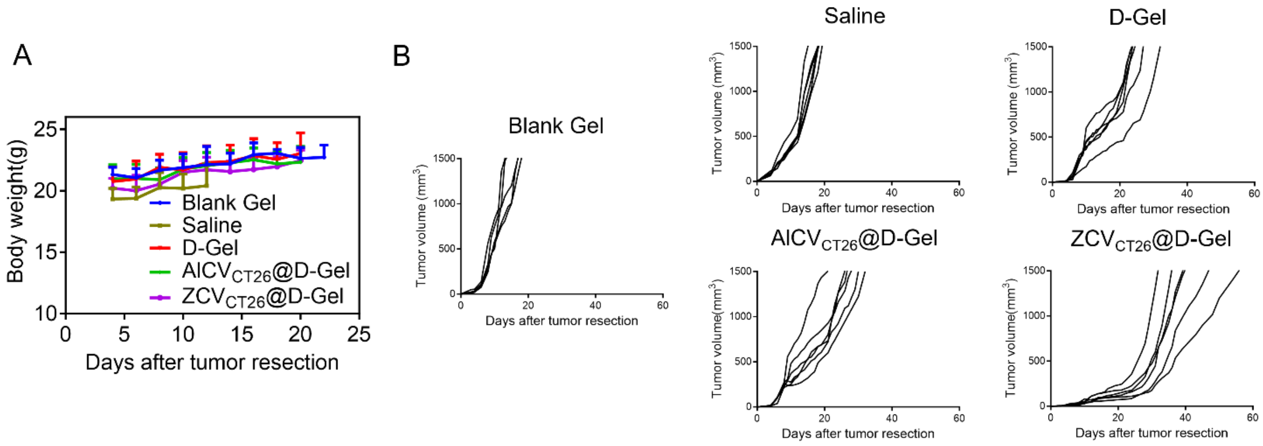

Figure S6. (A) The body weight of BALB/c mice, following single dose treatment with blank gel, saline, D-Gel, AlCV$_{CT26}$@D-Gel and ZCV$_{CT26}$@D-Gel. (B) Individual growth of tumor in orthotopic breast tumor recurrence models after incomplete tumor resection and local injection with various hydrogel formulations (n=5-6).

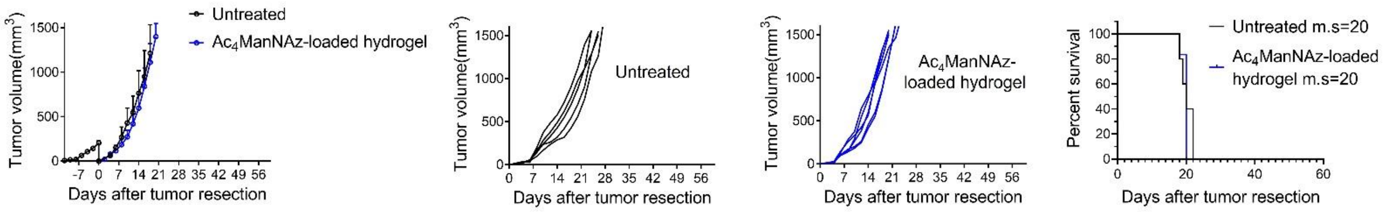

Figure S7. Tumor regrowth curves and animal survival of postsurgical 4T1 tumor models over time after treatment with Ac$_4$ManNAz-loaded hydrogel (n=5).

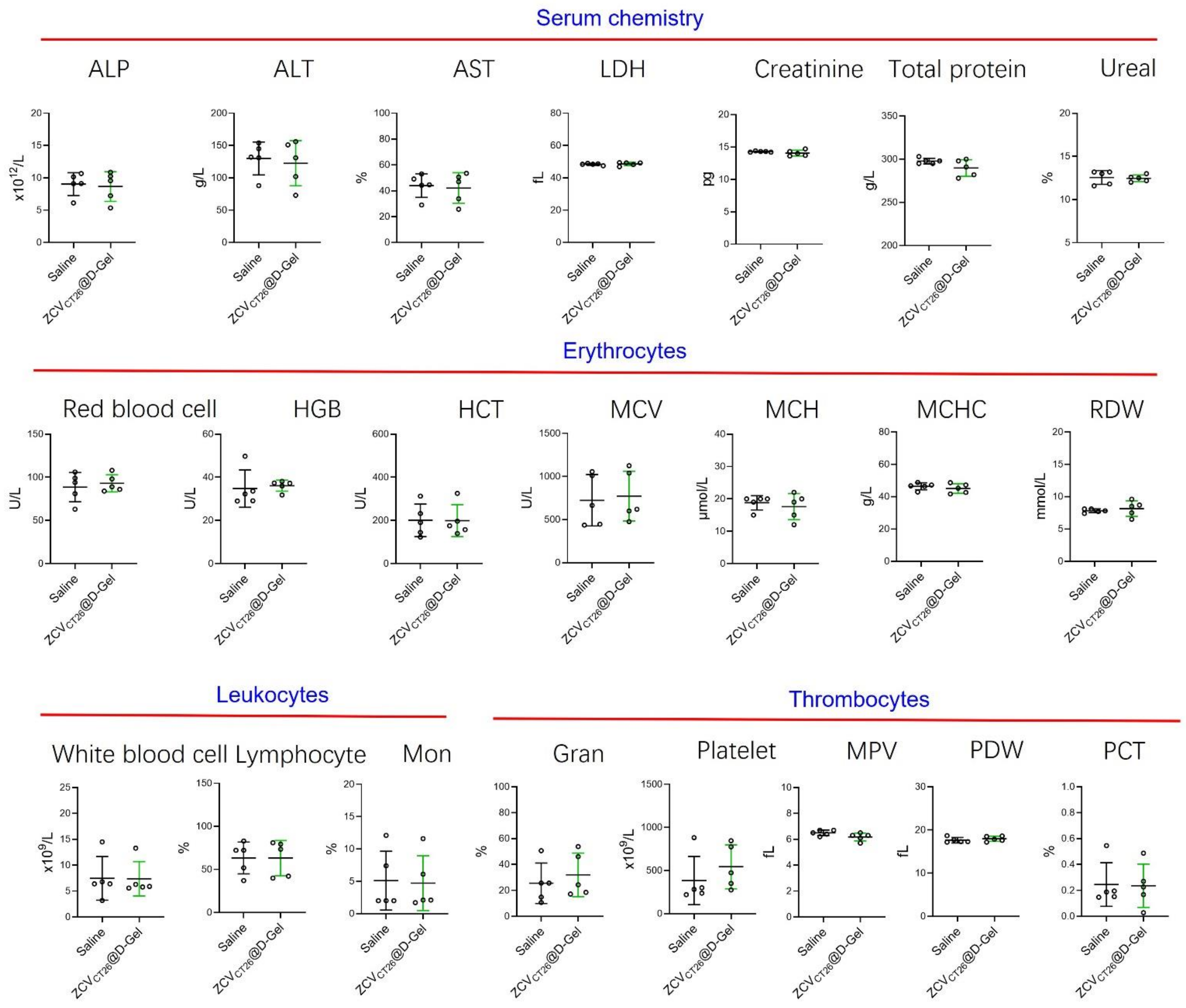

Figure S8. Serum chemistry, and hematological cell studies at endpoint after mice received different treatments. After tumor resection, mice randomly divided into Saline group and ZCV$_{CT26}$@D-Gel group, and blood were collected at endpoint on day 18 to analyze the serum chemistry, and hematological cell studies.

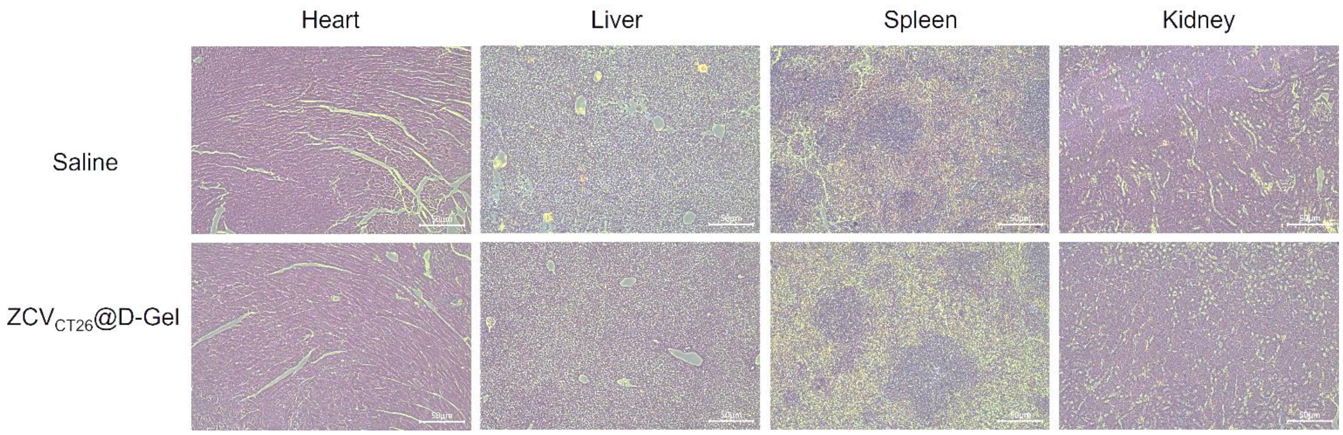

Figure S9. Hematoxylin and eosin staining of major organs (heart, liver, spleen, kidney) of postsurgical mice of 4T1 tumors receiving ZCV$_{CT26}$@D-Gel treatment for 18 days. Scale bar, 50 μm.

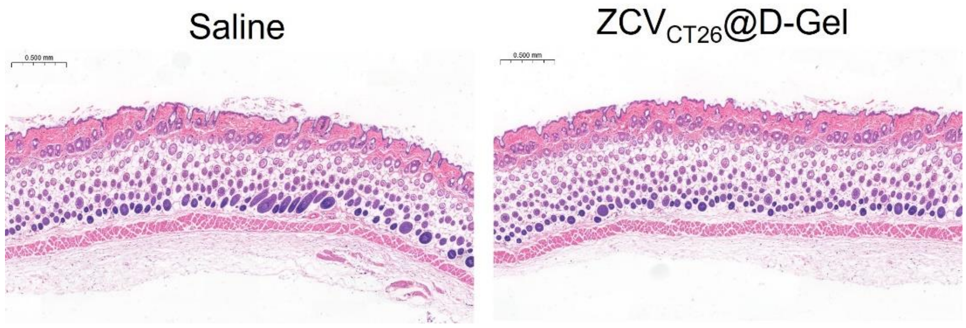

Figure S10. Hematoxylin and eosin staining of host skin tissues around ZCV$_{CT26}$@D-Gel 18 days post-injection. Scale bar, 500 μm.

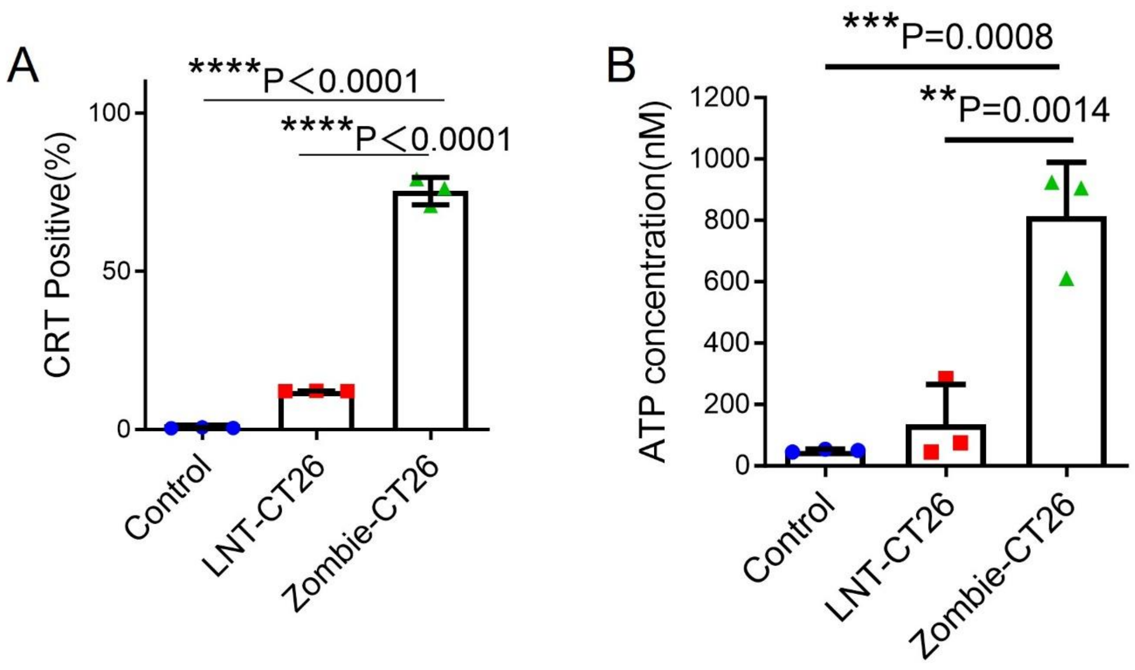

Figure S11. The DAMPs level of liquid nitrogen treated CT26 cells and zombie CT26 cells. CT26 cells were first treated with an oncolytic peptide of LTX-315 at 37 °C for 2 h, and incubated with DSPE-PEG$_{2k}$-DBCO at 37 °C for 20 min to obtain zombie-CT26. To obtain the LNT-CT26, CT26 cells were suspended in the cell cryopreservation medium and immersed in liquid nitrogen for 12 h and then thawed at 37 °C and washed with PBS thrice, followed by co-culturing with DSPE-PEG$_{2k}$-DBCO (100 μg/mL) at 37 °C for 20 min. After that, cells were washed with PBS and collected. Next, surface exposure of CRT (A) and extracellular secretion of ATP (B) were analyzed after LVT-CT26 and zCT26-DBCO were suspended in fresh culture medium for another 24 h.

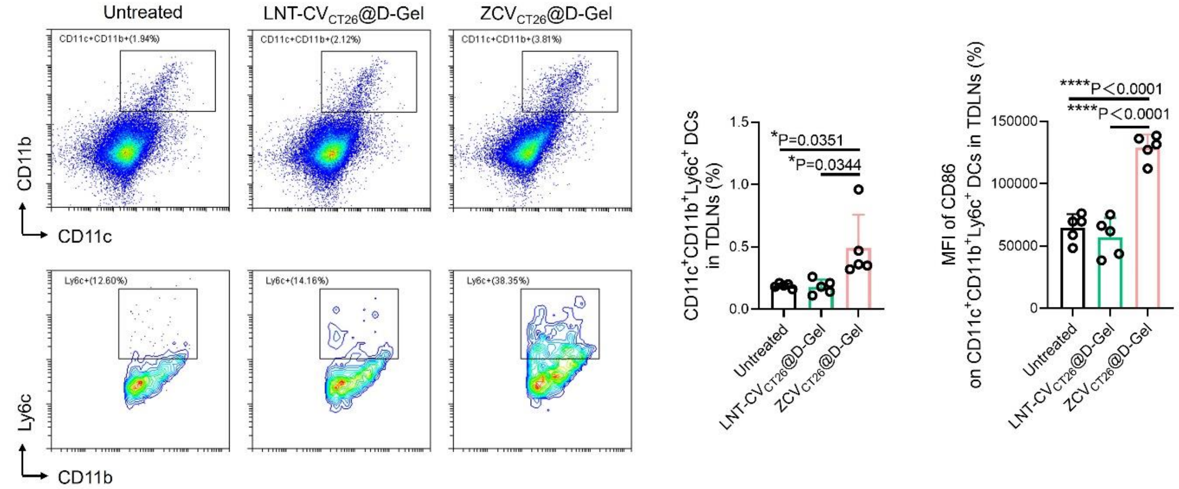

Figure S12. Flow cytometry analysis of $CD11c^+CD11b^+Ly6c^+$ DCs population in tumor-draining lymph node, and evaluation of their co-stimulatory CD86 level after postsurgical 4T1 tumor models were treated with LNT-CV_{CT26}@D-Gel and ZCV_{CT26}@D-Gel (n=5). Data are shown as mean ± SD; *p < 0.05, **p <0.01, ***P < 0.001, ****P < 0.0001.

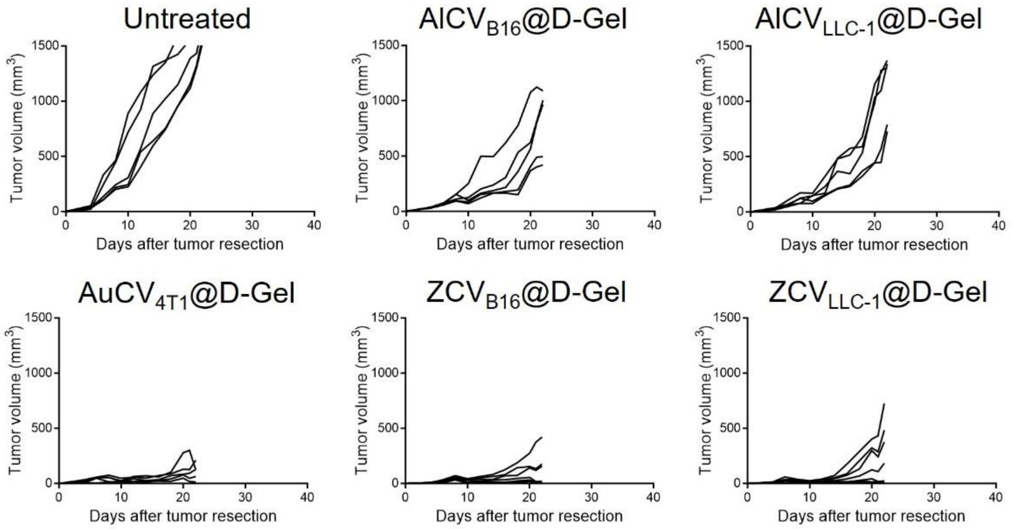

Figure S13. Individua growth curves of recurrent 4T1 breast tumors post-surgery, following single dose treatment with melanoma B16 cell-derived allogeneic vaccine (ZCV_{B16}@D-Gel, $AlCV_{B16}$@D-Gel), LLC-1 Lewis lung cancer cells-derived allogeneic vaccine (ZCV_{LLC-1}@D-Gel, $AlCV_{LLC-1}$@D-Gel), 4T1-derived autologous vaccine ($AuCV_{4T1}$@D-Gel) and untreated (n=5).

Reference

[1] Li, M.; Li, M.; Yang, Y.; Liu, Y.; Xie, H.; Yu, Q.; Tian, L.; Tang, X.; Ren, K.; Li, J.; Zhang, Z.; He, Q, *J. Controlled Release* **2020**, 321, 23−35.

[2] C. Wang, J. Wang, X. Zhang, S. Yu, D. Wen, Q. Hu, Y. Ye, H. Bomba, X. Hu, Z. Liu, Dotti, G.; Gu, Z, *Sci. Transl. Med.* **2018**, 10, eaan3682.

[3] M. Zhou, Q. Zuo, Y. Huang, L. Li, *Acta Pharm. Sin. B* **2022**, 12, 3383.
